# Dual-inhibition of NAMPT and PAK4 induces anti-tumor effects in 3D-spheroids model of platinum-resistant ovarian cancer

**DOI:** 10.1038/s41417-024-00748-w

**Published:** 2024-02-29

**Authors:** Kei Kudo, Yoshimi Endo Greer, Teruhiko Yoshida, Brittney S. Harrington, Soumya Korrapati, Yusuke Shibuya, Leah Henegar, Jeffrey B. Kopp, Takeo Fujii, Stanley Lipkowitz, Christina M. Annunziata

**Affiliations:** 1 Women’s Malignancies Branch, National Cancer Institute, National Institutes of Health, Bethesda, MD USA; 2 Department of Obstetrics and Gynecology, Division of Gynecology Oncology, Tohoku University School of Medicine, Miyagi, Japan; 3 Kidney Disease Section, Kidney Diseases Branch, National Institute of Diabetes and Digestive and Kidney Diseases, National Institutes of Health, Bethesda, MD USA; 4 Karyopharm Therapeutics, Newton, MA USA

**Keywords:** Ovarian cancer, Cell biology, Cancer stem cells

## Abstract

Ovarian cancer follows a characteristic progression pattern, forming multiple tumor masses enriched with cancer stem cells (CSCs) within the abdomen. Most patients develop resistance to standard platinum-based drugs, necessitating better treatment approaches. Targeting CSCs by inhibiting NAD+ synthesis has been previously explored. Nicotinamide phosphoribosyltransferase (NAMPT), which is the rate limiting enzyme in the salvage pathway for NAD+ synthesis is an attractive drug target in this pathway. KPT-9274 is an innovative drug targeting both NAMPT and p21 activated kinase 4 (PAK4). However, its effectiveness against ovarian cancer has not been validated. Here, we show the efficacy and mechanisms of KPT-9274 in treating 3D-cultured spheroids that are resistant to platinum-based drugs. In these spheroids, KPT-9274 not only inhibited NAD+ production in NAMPT-dependent cell lines, but also suppressed NADPH and ATP production, indicating reduced mitochondrial function. It also downregulated of inflammation and DNA repair-related genes. Moreover, the compound reduced PAK4 activity by altering its mostly cytoplasmic localization, leading to NAD+-dependent decreases in phosphorylation of S6 Ribosomal protein, AKT, and β-Catenin in the cytoplasm. These findings suggest that KPT-9274 could be a promising treatment for ovarian cancer patients who are resistant to platinum drugs, emphasizing the need for precision medicine to identify the specific NAD+ producing pathway that a tumor relies upon before treatment.

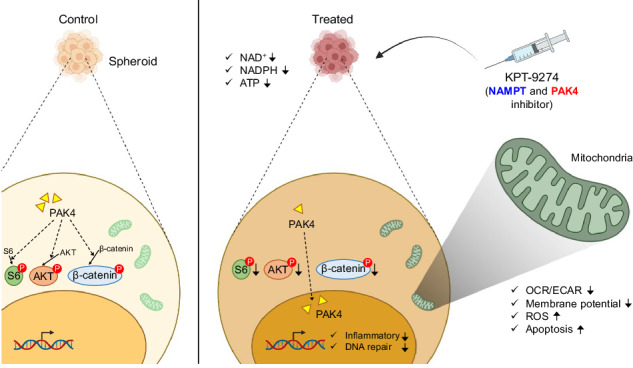

## Introduction

Ovarian cancer, which is the most lethal gynecological malignancy, is often diagnosed at late stages [1, 2]. Due to the difficulty of complete removal of the tumor in advanced stage, multidisciplinary treatment combining debulking surgery and chemotherapy with a platinum-based drug regimen is recommended [3, 4]. The platinum-based chemotherapy is efficacious in the majority of ovarian cancer patients, however, over 80% of advanced-stage cases relapse due to chemo-resistance, mandating treatment changes [5]. While vascular endothelial growth factor (VEGF) inhibitors, poly (ADP-ribose) polymerase (PARP) inhibitors, and immune checkpoint inhibitors have shown promise in some cases, the majority of patients eventually relapse, and thereby new treatment strategies are needed [3, 4, 6].

Ovarian cancer follows a unique metastatic pattern with floating tumor spheroid masses forming in the ascites and abdomen which lead to the metastasis and recurrence. These spheroid masses are enriched with cancer stem cells (CSCs) that are undifferentiated, self-renewal, highly tumorigenic, and drug-resistant [7]. The CSCs are enriched in 3D-cultured cells (spheroids) grown in non-adherent or ultra-low attachment cell-culture plates compared with conventional 2D-cultured cells, and the spheroids morphologically mimic the tumor mass in the ascites fluid [8]. Spheroids, which strongly reflect the characteristics of recurrent cancer are an effective preclinical model for predicting therapeutic efficacy against CSCs. Their use as models could lead to effective, novel therapeutic strategies for ovarian cancer patients.

**Fig. 1:**
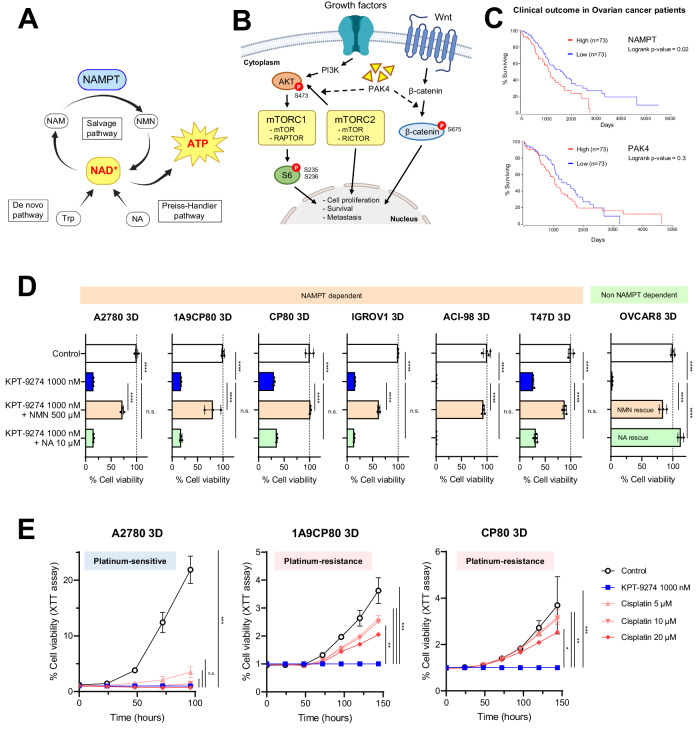
KPT-9274 is a potent and selective NAMPT inhibitor. **A** Schematic of the pathway for producing NAD^+^. **B** Schematic of the pathway related to PAK4, mTORC1, mTORC2, and Wnt/β-Catenin. **C** TCGA analysis revealed high expression of NAMPT in human ovarian cancer significantly correlates with worse prognosis. In PAK4, the correlation with worse prognosis is non-significant. **D** Ovarian cancer, endometrial cancer and breast cancer cell lines treated with KPT-9274 for 48 h at indicated doses. NMN or NA were added into media at indicated doses for confirming NMN rescue or NA rescue. (*n* = 3 or 4 independent experiments). **E** Cell viability with KPT-9274 or Cisplatin treatment in 3D-cultured A2780, 1A9CP80, and CP80 at indicated doses. Cell viability with KPT-9274 treatment was set to 1. 1A9P80 and CP80 are acquired resistance to Cisplatin treatment. (*n* = 4 independent experiments). Graph data were presented as mean ± SEM with *n* = 3 or 4 per group.

Nicotinamide adenine dinucleotide (NAD^+^) is an essential co-enzyme involved with metabolic processes required for survival and growth of all living cells. NAD^+^ is synthesized from three different pathways, including the Preiss–Handler pathway, generating NAD^+^ from nicotinic acid (NA) via nicotinic acid phosphoribosyltransferase (NAPRT); the de novo synthesis pathway, generating NAD^+^ from tryptophan (Trp) via quinolinate phosphoribosyl transferase (QPRT); and the salvage pathway, generating NAD^+^ from nicotinamide (NAM) via Nicotinamide phosphoribosyltransferase (NAMPT). Cells rely on the salvage pathway as the main sources of NAD^+^ [9–12] (Fig. 1A). NAMPT has been implicated in the pluripotency and dedifferentiation of CSCs, and several NAMPT inhibitors such as FK-866, GNE-617, GNE-618, CHS-828 have shown antitumor effects in a variety of cancers including colon cancer [13], gastrointestinal cancer [14], prostate cancer [15], breast cancer [16], and thyroid cancer [17].

KPT-9274 is a first-in-class, orally bioavailable NAMPT inhibitor designed to provide energy depletion, DNA repair inhibition, cell cycle arrest and growth inhibition [18] (Supplementary Fig. 1A). KPT-9274 targets two enzymes, NAMPT and p21 activated kinase 4 (PAK4). The expression level of PAK4 is often elevated in various types of cancers at DNA, RNA, or protein level, and is proposed as a diagnostic biomarker for cancer [18]. PAK4 phosphorylates β-Catenin, at serine 675, preventing its degradation and promoting cell proliferation [19] (Fig. 1B). PAK4 also boosts mTOR Complex 2 (mTORC2) kinase activity towards AKT at Ser473 [20, 21], which subsequently triggers mTORC1 activation [22]. The active mTORC1, composed of mTOR and RAPTOR, spurs cell proliferation through S6 ribosomal protein phosphorylation at Ser235/236 [23]. At present, KPT-9274 has been tested in clinical trial for refractory/relapsed hematologic tumors (NCT04914845). While its therapeutic efficacy has been demonstrated preclinically in various cancer types including hematologic malignancies [24, 25], breast cancer [26], and sarcoma [27], primarily in 2D-cultured cells, the potential of KPT-9274 on ovarian cancer cell lines remains unexplored. Moreover, the impact of KPT-9274 on ovarian cancer cell lines remains unverified. In this study, we addressed these issues with platinum-resistant 3D-cultured spheroids as a preclinical model. The findings indicate that KPT-9274 curbs mitochondrial function and triggers cell apoptosis via PAK4 kinase inhibition in an NAD^+^-dependent manner, suggesting a new potential therapy for ovarian cancer patients.

## Materials and methods

### Antibodies and chemicals

The following primary antibodies were purchased from Cell Signaling Technology (MA, USA) and used at the indicated dilution for Western blot analysis: IFNGR1 (#34808 S, 1:1000), IFIT1 (#14769 S, 1:1000), IFITM1 (#13126 S, 1:1000), IFITM2/3 (#96156 S, 1:1000), PBEF/NAMPT (#86634 S, 1:1000), RAPTOR (#2280 S, 1:1000), S6 Ribosomal protein (#2217 S, 1:1000), phospho-S6 Ribosomal protein (S235/236) (#4858 S, 1:1000), AKT (#9272 S, 1:1000), phospho-AKT (S473) (#9271 S, 1:1000), phospho-β-Catenin (S675) (#9567 S, 1:1000), Lamin B1 (#12586 S, 1:1000). HSP90α/β (#sc-13119, 1:1000) antibody was obtained from Santa Cruz Biotechnology (TX, USA). PAK4 (#14685-1-AP, 1:1000) and GAPDH (#60004-1-Ig, 1:1000) antibodies were purchased from Proteintech (IL, USA). β-catenin (#c19220, 1:1000) antibody was obtained from BD Biosciences (CA, USA). Poly (ADP-ribose) (#10407, 1:2000) antibody was obtained from Immuno-Biological Laboratories (Gunma, Japan). The following secondary antibodies were purchased from LI-COR Biosciences (NE, USA): IRDyeTM 800CW (#926-32210 anti-mouse or #926-32211 anti-rabbit, 1:5000), IRDyeTM 680RD (#926-68070 anti-mouse or #926-68071 anti-rabbit,1:5000). The following compounds were purchased from the indicated suppliers for in vitro studies: Cisplatin (#S1166, Selleck, TX, USA), FK-866 (#HY-50876, MedChemExpress, NJ, USA), GNE-617 (#HY-15766, MedChemExpress), β-Nicotinamide mononucleitide (NMN) (#N3501-25MG, Millipore Sigma, MO, USA), and Nicotinic acid (NA) (#N4126-100G, Millipore Sigma). PAK4-NAMPT dual inhibitor (KPT-9274), the anti-tumor drug of focus used in this study, was kindly provided from Karyopharm Therapeutics (Newton, MA, USA).

### Cell lines and tissue culture

Ovarian cancer cell lines (A2780, IGROV1, OVCAR8, and SKOV3), endometrioid cancer cell lines (EFE-184 and KLE) were purchased from the American Type Culture Collection (ATCC, VA, USA) and ACI-98 was kindly provided by Carrie D. House (San Diego State University). Breast cancer cell lines (T47D and MCF-7) were kindly provided by Stanley Lipkowitz. Ovarian cancer cell lines (1A9CP80 and CP80) were kindly provided by Antonio Tito Fojo (Columbia University). Cells were cultured at 37 °C in a 5% CO_2_ environment. For 2D-cultured cells, RPMI 1640 (#11875093, Thermo Fisher Scientific, USA)　medium supplemented with 10% fetal calf serum (FCS) (#100-106, GeminiBio, CA, USA), penicillin (100 units/mL) and streptomycin (100 units/mL) (#15140-122, Thermo Fisher Scientific) was used. For 3D-spheroids, ultra-low attachment plates (Corning, NY, USA) were used with Stem Cell culture Media, consisting of 1% KnockOut serum replacement (#10828-010, Thermo Fisher Scientific), 1% penicillin/streptomycin, 0.1% Insulin-Transferrin-Selenium (#41400-045, Thermo Fisher Scientific) and 0.4% Bovine Serum Albumin (#A9418, Millipore Sigma). Cultures were grown for 3 days prior to drug experiments. Mycoplasma infection was addressed using Plasmocin^TM^ prophylactic (#ant-mpp, InvivoGen, CA, USA) treatment, with confirmation of its absence.

### Western blotting

Cells were rinsed with PBS and lysed using the 0.5% NP-40 (#13021, Millipore Sigma) with Halt^TM^ protease and phosphatase inhibitor cocktail (#78442, Thermo Fisher Scientific). Cytoplasmic and nuclear lysate were prepared using a Rapid, Efficient And Practical (REAP) method [28]. Briefly, cell pellets were resuspended in ice-cold 0.5% NP-40 in PBS and centrifuged at 4 °C for 10 s (10,000 rpm). The supernatant was removed as cytoplasmic lysate. After the remaining supernatant was removed, the pellet was resuspended in 1 ml of ice-cold 0.5% NP-40 in PBS and centrifuged as above for 10 s and the supernatant was discarded. The pellet was resuspended in 0.5% NP-40 in PBS and designated as nuclear lysate. The BCA method (#23227, Thermo Fisher Scientific) was used for protein quantification. Lysates were boiled for 5 min, resolved using NuPAGE 4–12% SDS–PAGE gels (#NP0335BOX, Thermo Fisher Scientific) and transferred to NC membranes (#IB23002, Thermo Fisher Scientific) using iBlot2^TM^ Blotting System (#IB21001, Thermo Fisher Scientific). Membranes were blocked using Intercept^TM^ Blocking Buffer (#927-60001, LI-COR Biosciences), probed with primary antibodies overnight at 4 °C, and secondary antibodies at room temperature (RT) for 1 h. The immune complexes were visualized using the Odyssey^TM^ Fc Imager (LI-COR Biosciences).

### Cell viability assay

Cells were seeded at varying densities in 96-well plates. After 72 h, cell viability was measured with XTT assay (#11465015001, Millipore Sigma). Readings were normalized to the median of vehicle treated control wells and analyzed using microplate spectrophotometer SpectraMax i3 (Molecular Devices, CA, USA).

### NAD^+^, NADPH, and ATP concentration measurement

NAD^+^ levels were assessed using NAD^+^/NADH Quantification Colorimetric Kit (#K337-100, Biovision), NADPH levels were assessed using NADPH Quantitation Fluorometric Assay Kit (#K349-100, Biovision), and ATP levels were assessed using ATP Colorimetric/Fluorometric Assay Kit (#K354-100, Biovision) following the protocols provided by the supplier. Results were normalized with protein concentration and measured using SpectraMax i3.

### Seahorse XF assays

Cells were cultured in 96-well ULA plates (#7007, Corning, NY, USA) with Stem Cell culture Media containing 0.2 mM Matrigel^TM^ (#354230, Corning). Slow-growing cells (SKOV3, EFE-184, KLE, MCF-7, 1A9CP80 and CP80) were plated at 2000 cells/well, while fast-growing cells (A2780, IGROV1, OVCAR8, ACI-98, and T47D) were plated at 1000 cells/well. After 72 h, cells were treated with reagents for 48 h. Spheroids were then moved to XFe96 Spheroid Microplates (#102978-100, Agilent) in 10 replicates and incubated with 175 μL of serum-free unbuffered Seahorse XF RPMI Medium pH 7.4 with 1 mM HEPES (#103576-100, Agilent) pre-warmed at 37 °C and supplemented with 10 mM glucose, 2 mM glutamine and 1 mM Pyruvate (for analysis of mitochondrial oxidative metabolism) in a CO_2_-free incubator at 37 °C for 1 h. Cartridges equipped with oxygen- and pH-sensitive probes were preincubated with calibration solution (#100840-000, Agilent) overnight at 37 °C in a CO_2_-free incubator. The XFe96 Analyzer (Agilent) automatically mixed the assay media in each well for 15 min to allow the oxygen partial pressure to achieve oxygen equilibrium. Oxygen consumption rate (OCR) and extracellular acidification rate (ECAR) were assessed over time, before and after injecting compounds from Seahorse XF Cell Mito Stress Test Kit: Oligomycin (1 μM), FCCP (2 μM), and Rotenone+Antimycin (0.5 μM each). 25 μL of each compound was added to injection ports. OCR and ECAR values were adjusted based on spheroid size. Data points for OCR and ECAR represented average rates during measurement cycles and were reported as absolute rates (pmol/min for OCR, mpH/min for ECAR).

### GAPDH-mediated reaction quantification

GAPDH-mediated reaction was determined using a Glyceraldehyde 3 Phosphate Dehydrogenase Activity Assay Kit (#ab204732, Abcam, Cambridge, UK) according to the manufacturer’s instructions. Briefly, Reaction mix was added to the extracted samples and incubated at 37 °C for 30 min. NADH levels reflecting GAPDH-mediated responses were quantified by measuring absorbance (OD = 450). Results were normalized with protein concentration and measured using SpectraMax i3.

### Tetramethylrhodamine, Methyl Ester, Perchlorate (TMRM) intensity measurement

TMRM (#T668, Thermo Fisher Scientific) intensity (excitation/emission, 548/574 nm) was measured according to the manufacturer’s instructions Briefly, cells were added with staining solution at a final concentration of 100 nM and incubated for 30 min at 37 °C. After washing with PBS, the fluorescence was measured using SpectraMax i3. Results were normalized to the number of cells.

### MitoSOX^TM^ Red intensity measurement

MitoSOX™ Mitochondrial Superoxide Indicators (#M36008, Thermo Fisher Scientific) intensity (excitation/emission, 510/580 nm) was measured according to the manufacturer’s instructions. Briefly, cells were added with staining solution at a final concentration of 1 µM and incubated for 30 min at 37 °C. After washing with PBS, the fluorescence was measured using SpectraMax i3. The values were normalized with the cell viability by using CellTiter-Glo^TM^ Luminescent Cell Viability Assay (#G7570, Promega, WI, USA) in the same wells following the protocol provided with the assay kit.

### Cleaved caspase-3/7 quantification with IncuCyte^TM^ S3

Cells were seeded in 96-well ULA plates (#7007, Corning) with Stem Cell culture Media including 0.2 mM Matrigel^TM^ (#354230, Corning) (CP80: 2000 cells/well; ACI-98: 1000 cells/well), and after 72 h, the cells were treated with reagents at indicated doses adding IncuCyte^TM^ Caspase-3/7 Green Dye (#4440, Sartorius, Göttingen, Germany). The green mean intensity of spheroids was monitored quantitatively by IncuCyte^TM^ S3 (Sartorius).

### Cleaved caspase-3/7 luminescence measurement

Cell viability, cytotoxicity and apoptosis events in the same well were measured using ApoTox-Glo™ Triplex Assay Kit (#G6320, Promega) with or without Z-VAD-FMK (#S7023, Selleckchem, TX, USA). GF-AFC substrate was used to detect live-cells and bis-AAF-R110 substrate was used simultaneously to measure dead-cell protease activity. Luciferin, a substrate of luciferase, was measured to quantify cleaved caspase-3/7, an important indicator of apoptosis. In the experiment with Z-VAD-FMK, cells were pre-treated for 1 h at its final concentration of 20 μM, prior to KPT-9274 treatment.

### The Cancer Genome Atlas (TCGA) data preparation and integration

Ovarian cancer genomic and clinical data were obtained from TCGA portal. The results shown here are based upon data generated by the TCGA Research Network: https://www.cancer.gov/tcga. Patients with high and low expression groups were identified for NAMPT and PAK4 (Lower percentile = 25% (*n* = 73), Upper percentile = 25% (*n* = 73)). Transcripts Per Million (TPM) were acquired using patient TCGA barcode IDs.

### RNA isolation and RNA-seq in ovarian cancer cell line

mRNA was extracted from 3D-cultured CP80 using the RNeasy^TM^ Plus Mini Kit (#74134, Qiagen, Venlo, Netherlands). Total RNA samples were sequenced at the Frederick National Laboratory for Cancer Research sequencing facility, National Cancer Institute. Four control and four KPT-9274 treated mRNA-seq samples were sequenced on NextSeq 2000 P2 with Illumina Stranded mRNA Ligation Kit and paired-end sequencing. Samples yielded 118 to 137 million pass filter reads, over 95% with Q30 quality score. After trimming using Cutadapt (version 1.18) [29], reads were aligned to hg38 reference genome and transcripts with STAR (version 2.7.0 f) [30]. STAR/RSEM tools quantified gene expression, calculating normalized TPM count. Data were stored in NCI Data Vault for long-term security.

### Data analysis in RNA-seq

To identify Differentially Expressed Genes (DEGs), heatmap and volcano plot were created with Qlucore omics explorer (ver. 3.8). For identification of the functions and relevant pathways of DEGs, enrichment analysis and Ingenuity^TM^ Pathway Analysis (IPA^TM^, QIAGEN) were conducted. Enrichment analysis used Hallmark gene sets and Kyoto Encyclopedia of Genes and Genomes (KEGG) pathways from Gene Set Enrichment Analysis (GSEA) software.

### Immunofluorescent staining in spheroids

Spheroids were fixed using 3.7% formaldehyde at 4 °C for 48 h, then permeabilized with 100% methanol at RT for 30 min. After washing with 0.1% TBST, blocking was performed for 30 min at RT. Primary antibody (phospho-S6 Ribosomal protein (S235/236) (#4858 S, 1:1000) and phospho-AKT (S473) (#9271 S, 1:1000)) diluted using 5% BSA in PBS with 10% Goat serum (#G9023-10ML, Millipore Sigma) was added, and spheroids incubated on a rotator at 4 °C for 24 h. Following repeating wash step, spheroids were exposed to secondary antibody (Alexa Fluor^TM^ 568 goat anti-rabbit IgG (H + L) (#A11036, 1:1000, Thermo Fisher Scientific) is for phospho-S6 Ribosomal protein and Alexa Fluor^TM^ 488 goat anti-rabbit IgG (H + L) (#A11034, 1:1000, Thermo Fisher Scientific) is for phospho-AKT (S473)) diluted using 5% BSA in PBS with 10% Goat serum with NucBlue^TM^ Fixed Cell Stain ReadyProbes^TM^ reagent (#R37606, 2 drops/ml, Thermo Fisher Scientific) on a rotator at 4 °C for 24 h.

### Confocal fluorescence microscopy

Spheroids were observed with a Nikon Eclipse Ti2 microscope with CSU-W1 SoRa confocal unit (Nikon, Tokyo, Japan). Spheroids were imaged with 20× or 40×WI objective with excitation wavelengths of 405, 488, and 561 nm used with 0.9 μm Z-slices. NIS-Elements AR (version 5.21.03) was used for image acquisition.

### AKT kinase activity measurement

Incucyte^TM^ Kinase AKT Green/Red Lentivirus (#BA-04868, Sartorius) was used to quantify AKT kinase activity by expressing a green fluorescent protein (GFP)-tagged AKT substrate sensitive to phosphorylation-dependent subcellular localization, alongside a red fluorescent protein (RFP)-tagged nuclear marker for boundary indication. Cells were seeded in growth medium at a density to achieve 15–35% confluence at the time of infection.at time of infection. Incucyte^TM^ Kinase Akt Lentivirus was added at MOI = 3 diluted in Opti-MEM^TM^ I Reduced Serum Medium. After incubation for 24 h, the medium was removed and replaced with fresh growth medium. To efficiently eliminate non-transduced cells, Blasticidin S HCl (#A11139-03, Thermo Fisher Scientific) was used at a final concentration of 0.5 μM for 3 days. Incucyte^TM^ Kinase AKT response was assessed using Nuclear Translocation Ratio (NTR), measuring green fluorescence in cytoplasm and nucleus with IncuCyte^TM^ S3. NTR is calculated as 1 - (Green Intensity in Red+Green Overlap / Green Intensity in Green). Quantitative analysis of Akt activity was performed in CP80 in 2D-culture due to technical difficulties in quantifying NTR in 3D-spheroids.

### RNA interference

For the short interfering RNA (siRNA) experiment, adherent cells to achieve 50–70% confluency on the following day were transfected at a final concentration of 30 nM with ON-TARGETplus^TM^ Human non-targeting siRNA (#D-001810-01-20, Dharmacon) or ON-TARGETplus^TM^ SMARTpool Human NAMPT-targeting siRNA (#L-004581-00-0005, Dharmacon) using Lipofetamine^TM^ RNAiMAX Regent (#13778-150, Thermo Fisher Scientific) and Opti-MEM^TM^ I Reduced Serum Medium (#11058-021, Thermo Fisher Scientific). On the following day, cells were trypsinized and seeded onto ultra-low attachment plates. 72 h following transfection, spheroids were harvested.

### Statistical analyses

Data shown are mean ± SEM. Statistical analyses were performed in GraphPad Prism 8 software.

Significance of differences was determined using Student’s *t*-test or One-way ANOVA for Tukey’s multiple comparisons test or Logrank test. *p < 0.05, **p < 0.01, ***p < 0.001, ****p < 0.0001

## Results

### KPT-9274 is a potent and selective NAMPT inhibitor

To investigate the relevance of NAMPT and PAK4 in ovarian cancers, we first examined TCGA datasets. High expression of NAMPT was correlated with a significant reduction in overall survival in human ovarian cancer, suggesting that high NAMPT expression may be a prognostic factor in ovarian cancer. Similar results were observed in cervical and endometrial cancers, but there was no significant difference in breast cancer. High PAK4 expression lacked significant negative prognosis in these cancers, although a trend towards worse outcomes existed in endometrial cancer. (Fig. 1C and Supplementary Fig. 1B).

To assess preclinical effectiveness of KPT-9274 in gynecological cancers, we tested the effect on cell viability of 3D-spheroids from 11 cell lines of different histologic subtypes. The cell lines we used in this study had varying degrees of sensitivity to KPT-9274, and differed in their ability to be rescued by NMN or NA addition (Table 1). Based on manufacturer’s recommended concentrations and previous reports, KPT-9274 was tested up to 1000 nM as the highest concentration [24–27, 31]. The efficacy of KPT-9274 was demonstrated against A2780, 1A9CP80, CP80, IGROV1 and OVCAR8 in ovarian cancer, ACI-98 in endometrial cancer and T47D in breast cancer with IC_50_ 25–83 nM. In contrast, KPT-9274 did not inhibit the viability of SKOV3, EFE-184, KLE and MCF-7 at the highest dose, indicating NAD^+^ synthesis independent from NAMPT in these cells (Supplementary Fig. 1C). As expected, addition of NMN (downstream of NAMPT) rescued KPT-9274 impact across all cell lines (NMN rescue). To further test whether the cell lines produced NAD^+^ from NA by other pathways, rescue experiments were performed. We observed that NA, but not NMN, rescued the cytotoxic effect of KPT-9274 in OVCAR8 (NA rescue) (Fig. 1D and Supplementary Fig. 1D). Notably, the NAD^+^ production pathway differed across cell lines, suggesting biomarker analysis might be necessary to clarify the pathway involved before clinical application of KPT-9274 (Supplementary Fig. 1E).

**Table 1:** A list of cell lines used in this study and a summary of the results for each cell line.

	Diesease	Subtype	Character	KPT-9274 IC_50_ without NA In 3D cell culture	NMN rescue Yes or Not	NA rescue Yes or Not
A2780	Ovarian Epithelial Tumor	Endometrioid Carcinoma		80.0 nM	Yes	Not
1A9CP80	Ovarian Epithelial Tumor	Endometrioid Carcinoma	Cisplatin resistance	80.2 nM	Yes	Not
CP80	Ovarian Epithelial Tumor	Endometrioid Carcinoma	Cisplatin resistance	83.9 nM	Yes	Not
IGROV1	Ovarian Epithelial Tumor	Endometrioid Carcinoma		71.9 nM	Yes	Not
OVCAR8	Ovarian Epithelial Tumor	High-Grade Serous Carcinoma		43.0 nM	Yes	Yes
SKOV3	Ovarian Epithelial Tumor	Serous Carcinoma		>1000 nM	N/A	N/A
ACI-98	Endometrial Carcinoma	Endometrioid Carcinoma		52.1 nM	Yes	Not
EFE-184	Endometrial Carcinoma	Endometrioid Carcinoma		>1000 nM	N/A	N/A
KLE	Endometrial Carcinoma	Endometrioid Carcinoma		>1000 nM	N/A	N/A
T47D	Breast Carcinoma	Invasive Ductal Carcinoma	ERpos HER2neg	25.6 nM	Yes	Not
MCF-7	Breast Carcinoma	Invasive Ductal Carcinoma	ERpos HER2neg	>1000 nM	N/A	N/A

Next, to examine KPT-9274’s potential in platinum-resistant ovarian cancers, we tested using different cell lines, including platinum-sensitive (A2780) and platinum-resistant sub-lines (1A9CP80 and CP80). Based on clinical studies that reported the blood concentration of cisplatin [32, 33], the maximum concentration of cisplatin in this experiment was set at 20 μM. KPT-9274 demonstrated similar anti-tumor effects to cisplatin on A2780 (Fig. 1E). Notably, we observed KPT-9274 was significantly more effective than cisplatin in 1A9CP80 and CP80. Therefore, KPT-9274 could be a promising treatment for ovarian cancer that has developed resistance to platinum-based therapies.

### KPT-9274 suppresses the production of NAD^+^, NADPH, and ATP

**Fig. 2:**
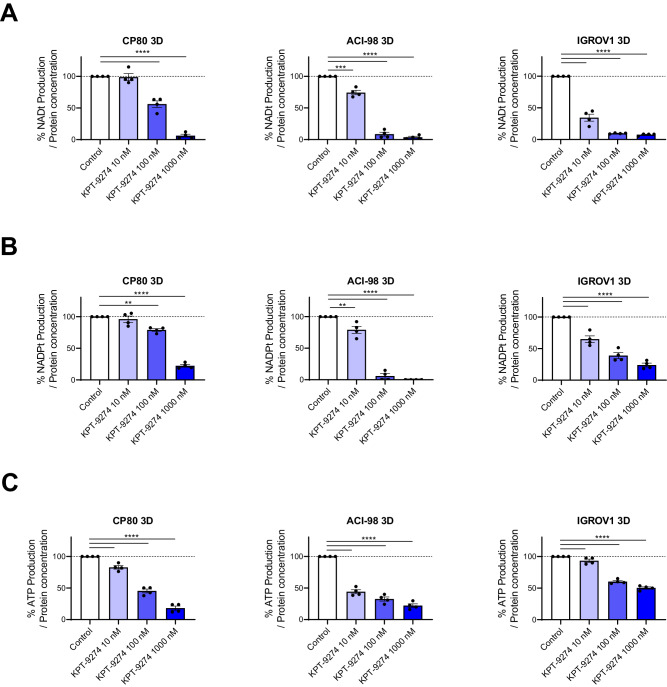
KPT-9274 suppressed the production of NAD^+^, NADPH, and ATP. **A** Change in total NAD levels in 3D-cultured CP80, ACI-98, and IGROV1 after treatment with KPT-9274 for 48 h at indicated doses relative to Control. (*n* = 4 independent experiments). **B** Change in total NADP levels in 3D-cultured CP80, ACI-98, and IGROV1 after treatment with KPT-9274 for 48 h at indicated doses relative to Control. (*n* = 4 independent experiments). **C** Change in total ATP levels in 3D-cultured CP80, ACI-98, and IGROV1 after treatment with KPT-9274 for 48 h at indicated doses relative to Control. (*n* = 4 independent experiments). Graph data were presented as mean ± SEM with *n* = 4 per group.

To assess KPT-9274 impact on NAMPT-dependent cell lines, we first measured NAD^+^ and NADPH production at various concentrations. Using 3D-cultured CP80, ACI-98, and IGROV1, KPT-9274 inhibited NAD^+^ and NADPH production in a dose-dependent manner (Fig. 2A, B).

To further investigate the mechanism, we next tested the effect of KTP-9274 on ATP production, as NAD^+^ is essential for ATP generation through glycolysis and the TCA cycle [34]. Consistent with the effect on NAD^+^ and NADPH production, KPT-9274 treatment significantly reduced ATP levels (Fig. 2C). Together, KPT-9274 is a selective NAMPT inhibitor that causes a multifaceted anti-tumor effect against NAMPT-dependent cell lines. It inhibits NAD^+^, NADPH, and ATP production, suggesting a comprehensive disruption of vital cellular processes.

### KPT-9274 causes the suppression of mitochondrial function

**Fig. 3:**
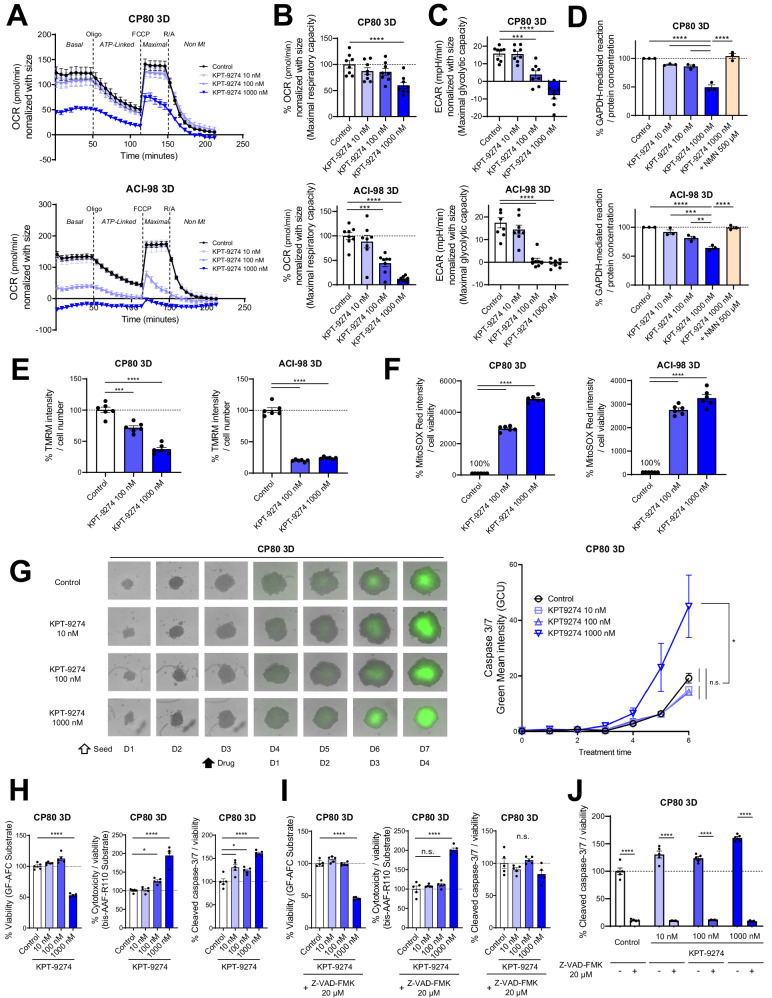
KPT-9274 causes the suppression of mitochondrial function. **A** Representative OCR pattern in 3D-cultured CP80 and ACI-98 as a function of time (min), normalized with spheroid size. The spheroids were treated with KPT-9274 for 48 h at indicated doses. Oligomycin (Oligo), carbonyl cyanide-4-(trifluoromethoxy)phenylhydrazone (FCCP), rotenone and antimycin A (R/A) were added to measure Basal OCR, ATP content, maximal OCR, and Non-mitochondrial OCR. (*n* = 8 independent experiments) Basal: Basal respiration, ATP-Linked: ATP-Linked Production, Maximal: Maximal respiration, Non Mt: Nonmitochondrial respiration. **B** Maximal respiratory capacity in OCR (*n* = 8 independent experiments). **C** Maximal glycolytic capacity in ECAR (*n* = 8 independent experiments). **D** Change in GAPDH-mediated reaction normalized by protein concentration in 3D-cultured CP80 and ACI-98 after treatment with KPT-9274 for 48 h at indicated doses relative to Control. NMN were added into media at indicated doses for confirming NMN rescue. (*n* = 3 independent experiments). **E** Change in TMRM intensity normalized with cell number in 3D-cultured CP80 and ACI-98 after treatment with KPT-9274 for 48 h at indicated doses relative to Control. (*n* = 6 independent experiments). **F** Change in MitoSOX^TM^ Red intensity normalized with cell viability in 3D-cultured CP80 and ACI-98 after treatment with KPT-9274 for 48 h at indicated doses relative to Control. (*n* = 6 independent experiments). **G** Left: Fluorescence analysis of CP80 spheroids after treatment with KPT-9274 at indicated doses. The spheroids were treated 3days after seeding cells. Time-dependent bright field and fluorescent overlay images of Cleaved caspase-3/7 for the spheroids. Right: Quantification of Green Mean Intensity as a function of time (days) using IncuCyte^TM^ S3. (*n* = 4 independent experiments). **H** Change in GF-AFC substrate intensity (left), bis-AAF-R110 substrate intensity normalized with viability (middle), and cleaved caspase-3/7 normalized with viability (right) in 3D-cultured CP80 after treatment with KPT-9274 for 96 h at indicated doses relative to Control. (*n* = 5 independent experiments). **I** Changes in treatment with Z-VAD-FMK 20 μM for 1 h before the same treatment as (**H**). (*n* = 5 independent experiments). **J** Comparison in cleaved caspase-3/7 normalized with viability in the absence and presence of prior Z-VAD-FMK. (*n* = 5 independent experiments). Graph data were presented as mean ± SEM with *n* = 3 or 4 or 5 or 6 or 8 per group.

Based on the inhibitory effect on NAD^+^, NADPH and ATP, we next hypothesized that KPT-9274 affects mitochondrial functions. Using the Mito Stress Test with XFe96, we assessed KPT-9274 impact on mitochondria function in 3D-cultured CP80 and ACI-98. As anticipated, KPT-9274 reduced oxygen consumption rate (OCR), an established measure of mitochondrial function [35], in CP80 and ACI-98 cells in 3D-spheroids, affecting both basal and maximal respiration (Fig. 3A,B). Interestingly, KPT-9274 significantly suppressed not only OCR, but also maximal extracellular acidification rate (ECAR), reflecting glycolysis (Fig. 3C). NAD^+^ is a co-enzyme in the reaction catalyzed by Glyceraldehyde 3-phosphate dehydrogenase (GAPDH), which is an enzyme essential for the conversion of glyceraldehyde-3-phosphate to 1,3-bisphosphoglyceric acid in glycolysis [36]. Hence, we hypothesized KPT-9274 inhibits GAPDH. As anticipated, KPT-9274 inhibited the GAPDH-mediated reaction, and adding NMN to the medium reversed the inhibition (Fig. 3D). These findings suggest KPT-9274 suppresses not only mitochondrial ATP production, but also glycolysis.

Furthermore, we investigated the impact of KPT-9274 on mitochondrial membrane potential, using TMRM, a fluorescent dye dye which accumulates in active mitochondria with intact potentials, whch emits a bright signal in healthy cells. KPT-9274 significantly suppressed TMRM in CP80 and ACI-98 cells in 3D-spheroids (Fig. 3E). Conversely, we observed up-regulated MitoSOX^TM^ Red which reflects reactive oxygen species (ROS) generated in mitochondria of live cells (Fig. 3F). Moreover, cleaved caspase-3/7 signal was monitored over time using IncuCyte^TM^ Caspase-3/7 Green Dye. KPT-9274 treatment significantly up-regulated green fluorescence intensity per area of spheroid, indicating caspase 3/7 activity was induced by KPT-9274 (Fig. 3G and Supplementary Fig. 2A). To evaluate whether KPT-9274 induces cell death, we quantified viability, cytotoxicity, and apoptosis induction using ApoTox-Glo™ Triplex Assay Kit, with or without a pan-caspase inhibitor, benzyloxycarbonyl-Val-Ala-Asp-fluoromethyl ketone (Z-VAD-FMK) [37], after 96 h of KPT-9274 treatment at varying doses. As anticipated, KPT-9274 suppressed cell viability and induced cytotoxicity as well as cleaved caspase-3/7 activity (Fig. 3H, J). Pre-treatment with Z-VAD-FMK inhibited only cleaved caspase-3/7 secretion, while having no considerable effect on cytotoxicity (Fig. 3I,J). These results suggest that caspase-3/7 activity is a part of anti-tumor effects of KPT-9274, but not entirely attributed to the cytotoxicity.

### NAMPT correlates with inflammatory gene expression and PAK4 is associated with DNA repair genes in ovarian cancer patients

**Fig. 4:**
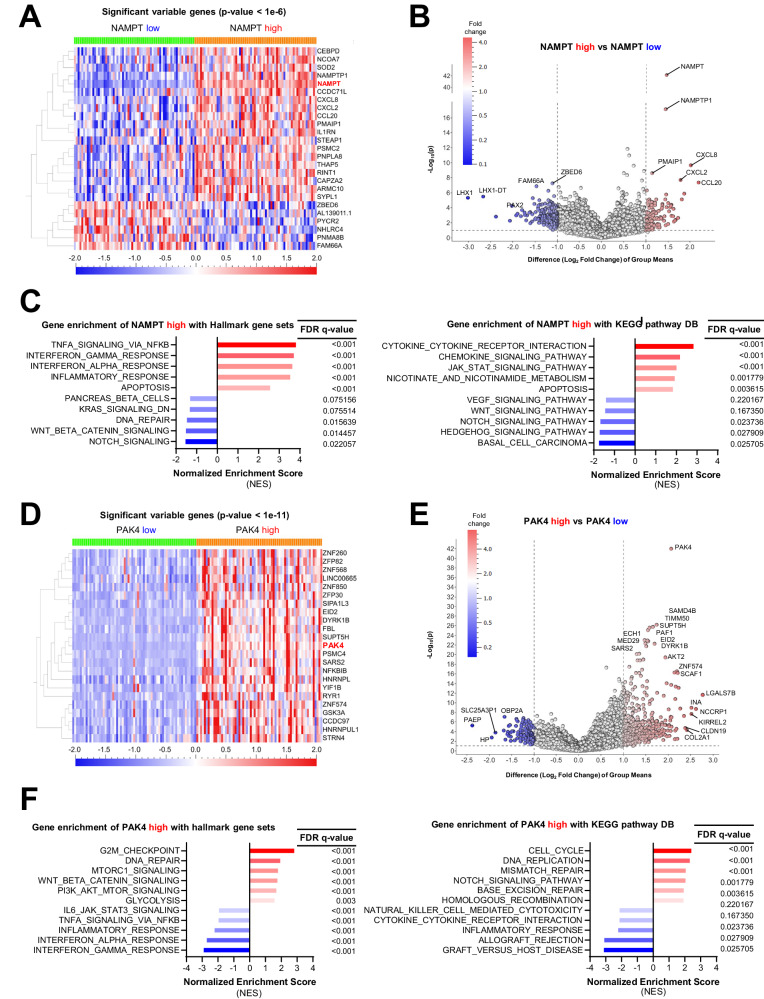
NAMPT correlates with inflammatory gene expression and PAK4 is associated with DNA repair genes in ovarian cancer patients. **A** Heat map shows the comparison of transcripts from the ovarian cancer tumors of NAMPT high patients and NAMPT low patients in different samples from TCGA. (Cutoff used: *p* < 1e-6). **B** Volcano plot showing distinct transcriptomic signatures in the NAMPT high and NAMPT low tumors. Volcano plot was generated to identify genes that were differentially enriched. (Cutoff used: |Difference (Log_2_ Fold Change) of group means | > 1, and -Log_10_ (*p*-value) > 1). **C** Normalized enrichment score of various gene sets in NAMPT high group using Hallmark gene sets in MSigDB and KEGG pathway DB are shown in bar plots. **D** Heat map shows the comparison with transcripts with the ovarian cancer tumors of PAK4 high patients and NAMPT low patients in different samples from TCGA. (Cutoff used: *p* < 1e-11). **E** Volcano plot showing distinct transcriptomic signatures in the PAK4 high and PAK4 low tumors. Volcano plot was generated to identify genes that were differentially enriched. (Cutoff used: |Difference (Log_2_ Fold Change) of group means | > 1, and -Log_10_ (*p*-value) > 1). **F** Normalized enrichment score of various gene sets in PAK4 high group using Hallmark gene sets in MSigDB and KEGG pathway DB are shown in bar plots.

To further characterize the impact of NAMPT and PAK4 in ovarian cancer, we evaluated the ovarian cancer RNA sequencing data from TCGA. We first compared patients with high and low NAMPT expression (Lower percentile = 25% (*n* = 73), Upper percentile = 25% (*n* = 73)) and developed a heatmap and volcano plot to detect DEGs (Fig. 4A,B). Top 20 DEGs between NAMPT high and low expression patients included NAMPT, NAMPTP1, ARMC10, CAPZA2, CXCL8, CCDC71L, NCOA7, PMAIP1, SYPL1, PNPLA8, CXCL2, CEBPD, CCL20, ZBED6, FAM66A, PNMA8B, PYCR2, PSMC2, SOD2, and STEAP1 (Supplementary Fig. 3A and Supplementary Table. 1). GSEA revealed that patients with high NAMPT expression exhibited enriched gene sets related to inflammation in hallmark gene sets and KEGG pathway database. The top five up-regulated gene sets in NAMPT-high patients were TNF-α signaling via NFκB, Interferon-γ response, Interferon-α response, and Apoptosis. Moreover, using KEGG pathway, the top five up-regulated gene sets were Cytokine-cytokine-receptor interaction, Chemokine signaling pathway, JAK-STAT signaling pathway, Nicotinate and nicotinamide metabolism, and Apoptosis (Fig. 4C and Supplementary Fig. 3B). Notably, the findings highlight a connection between high NAMPT expression and increased inflammation, suggesting the increased inflammation may contribute to a poorer prognosis in ovarian cancer patients.

Similarly, we next identified DEGs in patients with high and low PAK4 expression (*n* = 73, respectively). Results were visualized with a heatmap and volcano plot, highlighting 20 significant DEGs between PAK4 high and low expression patients including PAK4, POLR2I, ECH1, CAPNS1, PTOV1, RBPJ, ZNF628, YIF1B, KPNA5, RPL26P6, NDUFS7, ZNF865, ZC3H3, FRA10AC1, ZNF574, LY96, FRG1, MRPL2, C19orf47, and PPDPF (Supplementary Fig. 3C and Supplementary Table. 2). All 23 DEGs were highly expressed in high PAK4 patients (*p* < 1e-11) (Fig. 4D, E). GSEA revealed that the top five up-regulated gene sets were G2M checkpoint, DNA repair, mTORC1 signaling, Wnt/β-Catenin signaling, and PI3K-AKT-MTOR signaling in Hallmark gene sets. Moreover, top five up-regulated gene sets were Cell cycle, DNA replication, Mismatch repair, Base excision repair, and Homologous recombination in the KEGG pathway (Fig. 4F and Supplementary Fig. 3D). Collectively, these findings suggest elevated gene repair and cell proliferation functions in high PAK4 patients, potentially contributing to tumor cell survival and replication.

### KPT-9274 triggers suppression of inflammatory signaling

**Fig. 5:**
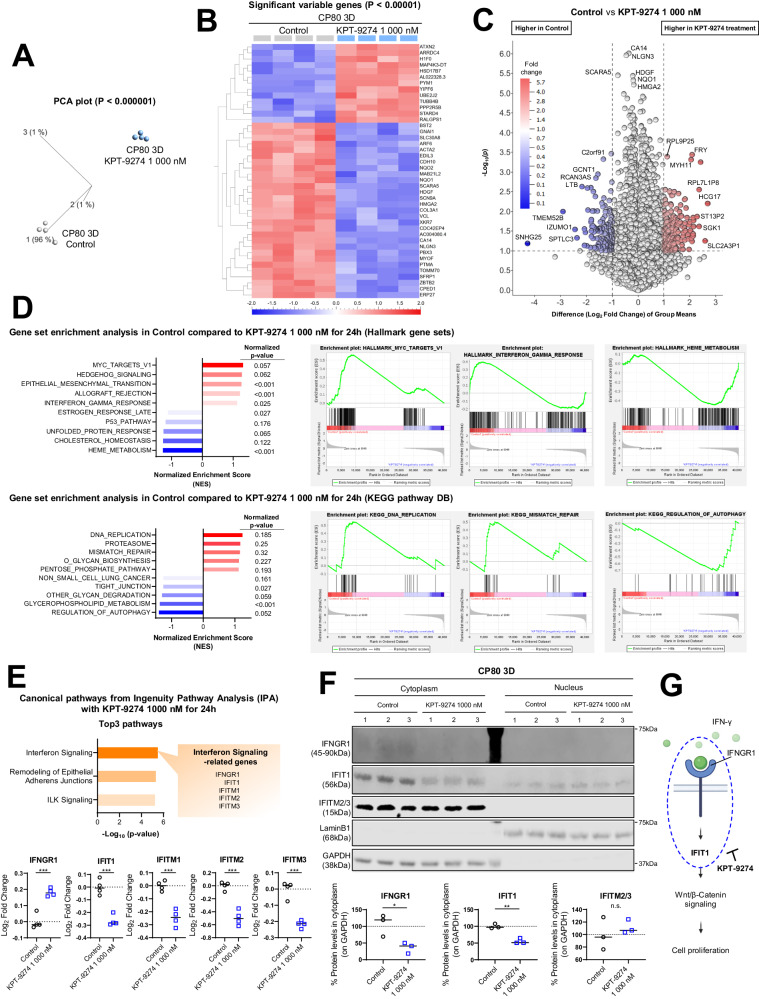
KPT-9274 triggers suppression of inflammatory signaling. **A** PCA showing gene profiles of 3D-cultured CP80 after treatment with KPT-9274 1000 nM for 24 h relative to Control. (Results shown are from four independent experiments). **B** Heatmap representing DEGs in treated 3D-cultured CP80 as described above. (Cutoff used: *p* < 1e-5). **C** Volcano plot generated to identify DEGs in 3D-cultured CP80 after KPT-9274 treatment relative to Control. (Cutoff used: |Difference (Log_2_ Fold Change) of group means | >1, and -Log_10_ (*p*-value) >1). **D** Left: Normalized enrichment score of various gene sets in Control group relative to KPT-9274 treatment are shown in bar plots. Right: GSEA in Control group relative to KPT-9274 treatment. (Top: Hallmark gene sets in MsigDB, bottom: KEGG pathway DB). **E** Top: Pathways affected by KPT-9274 treatment as identified by Ingenuity pathway analysis (IPA). Bottom: Normalized gene expression levels associated with Interferon Signaling in Control and KPT-9274 treatment. (*n* = 4 independent experiments). **F** Top: Immunoblotting for assessing the expression of IFNGR1, IFIT1, and IFITM2/3 in 3D-cultured CP80 cell lysates with KPT-9274 treatment at indicated doses. GAPDH and LaminB1 were shown as controls. (Left: cytoplasm lysate, Right: nuclear lysate) Bottom: Cytoplasmic protein levels normalized by GAPDH in Control and KPT-9274 treatment. (*n* = 4 independent experiments). **G** Schematic showing that KPT-9274 inhibits Wnt/β-Catenin signaling by reducing the expression of inflammatory-related proteins, including IFNGR1 and IFIT1. Graph data were presented as mean ± SEM with *n* = 4 per group.

We hypothesized that the anti-tumor effects of KPT-9274 arose from inhibition of gene expression related to inflammation, gene repair, and cell proliferation signaling. To validate this hypothesis, we performed RNA-seq analysis on 3D-cultured CP80 cells treated with DMSO (Control) or KPT-9274 for 24 h. First, principal component analysis (PCA) demonstrated that technical replicates in each group clustered together, indicating low variation between the replicate samples (Fig. 5A). Next, we conducted hierarchical clustering analysis to detect the DEGs based on RNA-seq data and constructed a heatmap and volcano plot to visualize the impact of KPT-9274 treatment. The top 20 DEGs between Control and KPT-9274 treatment were CA14, NLGN3, SCARA5, HDGF, NQO1, HMGA2, ERP27, HSD17B7, PPP2R5B, MYOF, PYM1, CDC42EP4, ACTA2, NQO2, YIPF6, ATXN2, PTMA, SLC30A8, SCN9A, and ZBTB2 (Supplementary Table 3). Interestingly, SNHG25, known for promoting ovarian cancer progression [38], and TMEM52B, associated with EGFR and E-cadherin modulation and tumor/metastasis suppression [39], significantly decreased with KPT-9274 treatment (Fig. 5B, C). Next, GSEA revealed the top five up-regulated gene sets in the Control compared to KPT-9274 treatment: Myc-targets-V1, Hedgehog signaling, Epithelial mesenchymal transition, Allograft rejection, and Interferon-γ in Hallmark gene sets. The up-regulated gene sets in KEGG pathway included DNA replication, Proteasome, Mismatch repair, O-glycan biosynthesis, and Pentose phosphate pathway (Fig. 5D). Our findings suggest that KPT-9274 regulates cell proliferation by suppressing the expression of these tumor growth-associated genes and pathways.

IPA revealed that KPT-9274 treatment suppressed the Interferon signaling pathway, Remodeling of epithelial adherens junctions, and ILK signaling. The genes linked to Interferon signaling, namely IFNGR1, IFIT1, IFITM1, IFITM2, and IFITM3, showed varying expression patterns upon treatment (Supplementary Fig. 4A, B). Specifically, IFNGR1, which encodes the IFN-γ receptor-1, was upregulated, while the others were downregulated (Fig. 5E). IFIT1 affects cancer cell behavior through Wnt/β-Catenin signaling [40], and IFITM1, IFITM2, and IFITM3 are related to antiviral functions [41]. To validate how these changes in transcriptomes affect protein expression, we tested expression of IFNGR1, IFIT1, IFITM1, IFITM2, and IFITM3 using Western blotting. IFITM1 was not detected (data not shown), and IFITM2/3 showed no significant differences between Control and KPT-9274 treatment. Interestingly, contrary to RNA-seq data, KPT-9274 significantly suppressed IFNGR1 expression, a membrane surface protein. Given that the protein is the functional component of IFNGR1, not the transcript, we concluded that the inhibition of IFNGR1 protein expression by KPT-9274 treatment observed in this experiment contributes to the suppression of cell proliferation. Moreover, IFIT1 cytoplasmic expression was significantly down-regulated by KPT-9274 (Fig. 5F), suggesting that KPT-9274 downregulates Wnt/β-Catenin pathway via a suppression of IFNGR1 and IFIT1, contributing to the anti-tumor effects (Fig. 5G).

### KPT-9274 down-regulates multiple kinase activities in the cytoplasm through a localization change of PAK4

**Fig. 6:**
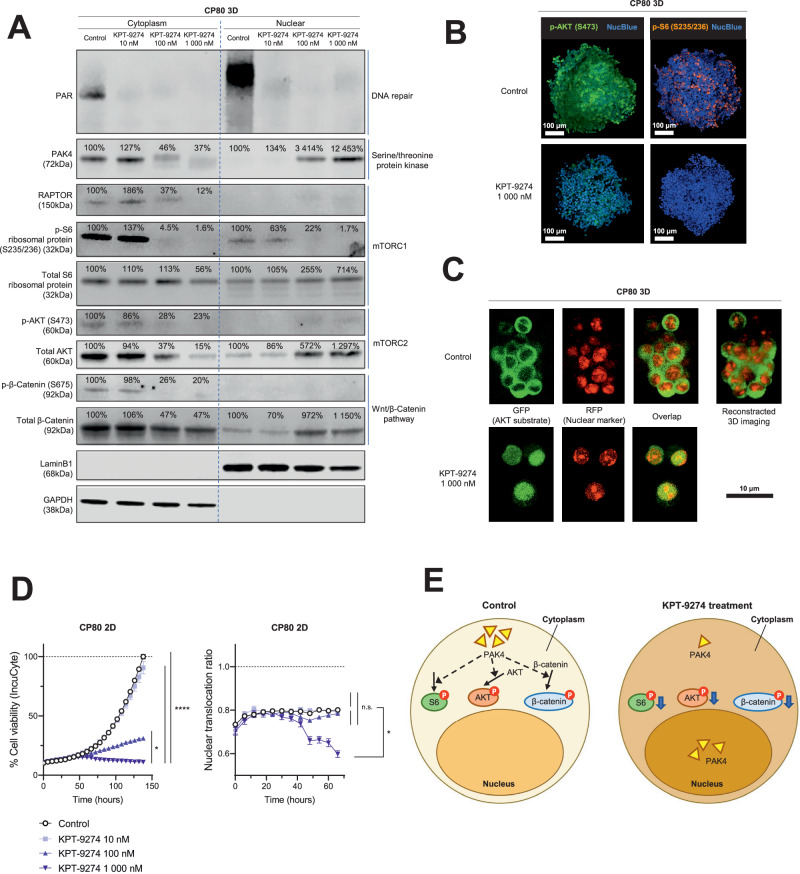
KPT-9274 inhibited cell proliferation by down-regulating kinase activity in the cytoplasm through a localization change of PAK4. **A** Immunoblotting for assessing the activity of DNA repair, Serine/threonine protein kinase, mTORC1, mTORC2, and Wnt/β-Catenin signaling in 3D-cultured CP80 cell lysates with KPT-9274 treatment at indicated doses. PAR for NAD^+^-mediated DNA repair, PAK4 for Serine/threonine protein kinase, RAPTOR and the phosphorylation of S6 (p-S6) at S235/236 for mTORC1, the phosphorylation of AKT (p-AKT) at S473 for mTORC2, and the phosphorylation of β-Catenin (p-β-Catenin) at S675 for Wnt/β-Catenin signaling. Total S6, AKT, β-Catenin, GAPDH, and LaminB1 were shown as controls. (Left: cytoplasm lysate, Right: nuclear lysate). **B** Representative images of CP80 spheroids after treatment with KPT-9274 1000 nM for 48 h relative to Control. (Results shown are from three independent experiments.) The spheroids were stained with phosphorylated S6 at S235/236 (orange), phosphorylated AKT at S473 (green), and NucBlue^TM^ (blue). Scale bars, 100 μm (low magnification). **C** 3D-cultured CP80 stably expressing the Incucyte^TM^ Kinase Akt Green/Red Indicator were treated with KPT-9274 1000 nM for 48 h. The image panel shows green fluorescence channel on the left, red fluorescence channels in the middle, and overlap channel on the right. Scale bars, 10 μm (high magnification). **D** Left: The kinetic graph shows cell proliferation in 2D-cultured CP80 with KPT-9274 treatment at indicated doses as a function of time (hours) using IncuCyte^TM^ S3. (Results shown are from six independent experiments.) Right: The kinetic graph shows 2D-cultured CP80 change in the Nuclear Translocation Ratio, which reflects translocation of the green fluorescent sensor from the cytoplasm to the nucleus, with KPT-9274 treatment at indicated doses as a function of time (hours) using IncuCyte^TM^ S3. (*n* = 6 independent experiments). **E** Schematic showing that PAK4 reduction in the cytoplasm by KPT-9274 treatment regulates phosphorylation of AKT, S6, and β-Catenin. Graph data were presented as mean ± SEM with *n* = 6 per group.

It has been shown that PAK4 regulates β-Catenin phosphorylation and mTOR complex function [19–23]. Hence, suppressing PAK4 leads to reduced kinase activity of various proteins, such as AKT, that are controlled by mTOR complexes. To validate the effect of KPT-9274 on kinase activity, we evaluated the expression of PAK4-affected proteins with Western blotting using cytoplasm and nuclear lysate. We also evaluated Poly (ADP-ribose) (PAR), which reflects the function of DNA repair [42], because RNA-seq results suggested KPT-9274 inhibited DNA repair. As expected, PAR expression was suppressed in both cytoplasm and nucleus following KPT-9274 treatment, suggesting impaired DNA repair by KPT-9274. Notably, PAK4, which was mostly localized in the cytoplasm before treatment, migrated into the nucleus after KPT-9274 treatment. In parallel to the shift of the localization of PAK4, cytoplasmic expression level of RAPTOR, Phospho-S6 Ribosomal Protein (Ser235/236), Phospho-AKT (Ser473), and Phospho-β-Catenin (Ser675) was decreased (Fig. 6A). RAPTOR and Phospho-S6 Ribosomal Protein (Ser235/236) reflect mTORC1 function [23]. Similar protein suppression was observed in whole cell lysates of 3D-cultured A2780, ACI-98, and CP80 cells (Supplementary Fig. 5A, B). Next, using FK-866, the first-in-class NAMPT inhibitor, and GNE-617, a specific NAMPT inhibitor [43], we conducted a similar validation. Despite successfully inhibiting NAD^+^ production, the subcellular distribution of PAK4 remained unaltered with specific inhibition of NAMPT alone, while and the impact on key proteins like RAPTOR, S6 Ribosomal Protein, AKT, and β-Catenin was inconsistent, displaying distinct patterns between NAMPT inhibitors (Supplementary Fig. 6A, B). These findings highlight that the alteration of PAK4 localization seems to be specific to KPT-9274.

In support of these Western blotting findings, immunofluorescence confocal imaging of the spheroids also revealed the fluorescence intensity of Phospho-S6 Ribosomal Protein (Ser235/236) and Phospho-AKT (Ser473) in the 3D-spheroids was suppressed with KPT-9274 treatment (Fig. 6B). Phospho-β-Catenin (Ser675) was difficult to detect (data not shown). To assess kinase activity from different perspectives, IncuCyte^TM^ Kinase AKT Assay was performed. AKT phosphorylation moves the green sensor from nucleus to cytoplasm. Conversely, AKT inhibition retains the sensor in the nucleus [44]. Interestingly, KPT-9274 treatment maintained the green sensor in the nucleus, indicating suppressed AKT kinase activity (Fig. 6C). Nuclear Translocation Ratio, reflecting sensor movement [44], was reduced by KPT-9274 in a concentration-dependent manner, linked to inhibited cell proliferation (Fig. 6D). Overall, these findings suggested that KPT-9274 hindered cell proliferation by lowering cytoplasmic kinase activity through altering PAK4 localization (Fig. 6E).

### Suppression of PAK4-mediated kinase activity by KPT-9274 is NAD^+^ dependent

**Fig. 7:**
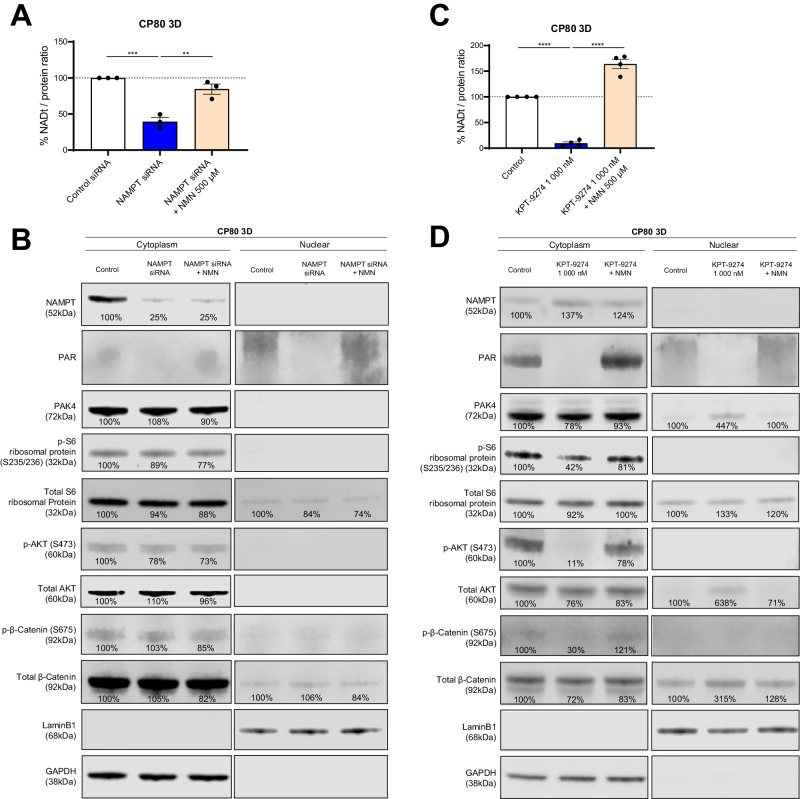
The suppressed PAK4-mediated kinase activity by KPT-9274 treatment is NAD^+^-dependent. **A** Change in total NAD levels in 3D-cultured CP80 with Control siRNA, NAMPT siRNA, and NAMPT siRNA plus NMN 500 μM. Spheroids were harvested 72 h after transfection. NMN was administered at the same time the trypsinized cells were seeded. (*n* = 3 independent experiments). **B** Immunoblotting for assessing the activity of NAMPT, DNA repair, Serine/threonine protein kinase, mTORC1, mTORC2, and Wnt/β-Catenin signaling in 3D-cultured CP80 cell lysates with Control siRNA, NAMPT siRNA, NAMPT siRNA plus NMN 500 μM. PAR for NAD^+^-mediated DNA repair, and the phosphorylation of S6 (p-S6) at S235/236 for mTORC1, the phosphorylation of AKT (p-AKT) at S473 for mTORC2, and the phosphorylation of β-Catenin (p-β-Catenin) at S675 for Wnt/β-Catenin signaling. Total S6, AKT, β-Catenin, GAPDH, and LaminB1 were shown as controls. (Left: cytoplasm lysate, Right: nuclear lysate). **C** Change in total NAD levels in 3D-cultured CP80 with Control, KPT-9274 1000 nM for 48 h, and KPT-9274 1000 nM plus NMN 500 μM for 48 h. (*n* = 4 independent experiments). **D** Immunoblotting for assessing the activity of NAMPT, DNA repair, Serine/threonine protein kinase, mTORC1, mTORC2, and Wnt/β-Catenin signaling in 3D-cultured CP80 cell lysates with Control, KPT-9274 1000 nM for 48 h, and KPT-9274 1000 nM plus NMN 500 μM for 48 h. The evaluated proteins are the same as those described in **B**. Graph data were presented as mean ± SEM with *n* = 3 or 4 per group.

To uncover whether the ability of KPT-9274 to suppress multiple kinase activities is a NAD^+^-dependent mechanism, we silenced NAMPT expression using siRNA. NAMPT-silenced cells showed approximately 60% less NAD^+^ content and about 75% less GAPDH corrected NAMPT expression than control siRNA-treated cells (Fig. 7A,B). Adding NMN to the medium had no effect on NAMPT expression, while rescued total NAD to 80% of the control. Consistent with the NAD^+^ production, PAR was suppressed in NAMPT-silenced cells and was rescued by NMN addition. However, NAMPT silencing did not impact PAK4, Phospho-S6 Ribosomal Protein (Ser235/236), Phospho-AKT (Ser473), and Phospho-β-Catenin (Ser675) (Fig. 7A,B). These findings suggest that reducing NAD^+^ through NAMPT silencing alone does not strongly suppress kinase activity.

Next, we tested whether supplemental NMN could rescue the kinase activity reduction caused by KPT-9274 treatment. As expected, KPT-9274 decreased NAD^+^ production by approximately 90% (Fig. 7C), while GAPDH corrected NAMPT expression increased (Fig. 7D), suggesting NAMPT upregulation due to NAD^+^ reduction. Importantly, NMN addition largely restored the suppressed PAK4, Phospho-S6 Ribosomal Protein (Ser235/236), Phospho-AKT (Ser473), and Phospho-β-Catenin (Ser675), indicating that suppressed PAK4-mediated kinase activity by KPT-9274 is NAD^+^-dependent (Fig. 7C,D). In conclusion, KPT-9274 demonstrated a promising activity against NAMPT or PAK4-driven cancer growth, suggesting it is a potential novel treatment for platinum-resistant ovarian cancer.

## Discussion

The majority of ovarian cancers recur due to resistance to platinum therapy, which is currently the first-line treatment in clinical practice [5]. While clinical biomarkers such as CA125, CA19-9, and CEA assist in monitoring disease status, their lack of specificity makes them unsuitable as therapeutic targets [1, 2]. This underscores the need to develop effective therapeutic strategies, including the identification of novel biomarkers [3, 4]. Given that NAD^+^ levels are elevated in cancer cells compared with non-malignant cells due to upregulated NAD^+^ biosynthesis [45], we report high NAMPT expression is associated with poor outcomes in ovarian cancers in TCGA data. Here, we used 3D-spheroids of ovarian cancer cell lines as model systems to mimic a CSC enriched tumor mass floating intraperitoneally, and found that KPT-9274 is a potential treatment option for platinum-resistant ovarian cancer, and NAMPT may serve as a prognostic and predictive biomarker and therapeutic target.

Our study also identified several novel mechanisms associated with the therapeutic effect of the dual inhibitor. We found that KPT-9274 inhibits mitochondrial function, depletes ATP, and induces caspase-3/7 activity (Figs. 2 and 3). Bioinformatic analysis of TCGA data indicated that NAMPT-high ovarian cancer patients had several elevated genesets involved with inflammatory response (Fig. 4A–C and Supplementary Fig. 3A,B), while PAK4-high ovarian patients had increased expression of genesets related to DNA replication/repair, mTORC1-signaling, Wnt-β-Catenin signaling, and PI3K-AKT signaling (Fig. 4D–F and Supplementary Fig. 3C,D). These findings were corroborated with the RNA-seq data comparing control 3D-spheroid and KPT-9274-treated spheroids. KPT-9274 inhibited multiple cellular mechanisms, including DNA replication/repair-related genes, interferon-gamma signaling (Fig. 5 and Supplementary Fig. 4). KPT-9274 also inhibited phospho-S6 ribosomal protein, phosphor-Akt, phosphor-β-catenin (Fig. 6A). We also found that KPT-9274 altered PAK4 localization to inhibit its kinase activity, distinct from conventional NAMPT inhibitors (FK-866, GNE-617) (Fig. 6 and Supplementary Fig. 6). However, the mechanism in which KPT-9274 alters intracellular localization of PAK4 from the cytoplasm to the nucleus remains to be determined. Nuclear PAK4 is shown to be correlated with poor prognosis in estrogen receptor α-positive breast cancer, and proposed as a novel predictive biomarker for bone metastasis [46]. Additional studies are required to delineate specific NAD^+^ biosynthesis pathway and PAK4 localization to provide more mechanistic insights into the anti-tumor effects of KPT-9274. Given the drug’s progression to clinical trials, we also need to keep in mind that the compound has potential off-target effects other than those of NAMPT and PAK4, which could affect other proteins and pathways. Interestingly, RNA-seq analysis revealed that KPT-9274 activated multiple pathways such as HEME metabolism, cholesterol homeostasis, regulation of autophagy, and glycerophospholipid metabolism (Fig. 5D). The implications of these pathway activations remain unknown at this time, and it is beyond our scope of the current study. Further investigation will be required to address the mechanisms of these potential off-target effects.

Interest in NAMPT as an anti-cancer target has led to the development of several NAMPT-specific inhibitors, including FK-866/APO-866, GNE-617, GNE-618, and CHS-828 [43, 47]. Studies has shown that NAMPT inhibition induces cytotoxicity in cancer cell lines, but not in non-cancer cells in vitro [48, 49]. In addition, the anti-tumor effect of NAMPT inhibitors in ovarian cancer has been demonstrated in vivo [12, 50]. Despite these successes, previous phase I or II clinical trials in various cancer types did not show an objective tumor remission and were halted due to substantial side effects [43]. As one of the reasons of the failure, it’s been indicated that some cancers are not NAMPT-dependent [51]. Given that the anti-tumor effect of NAMPT inhibitors can be reversed by NA supplemented in cell culture medium in NAPRT-dependent cancers. Therefore, NAMPT inhibitors may be ineffective for NAMPT-independent cancers in the clinic. Previous clinical trials tested KPT-9274 (NCT04914845 and NCT0272492) did not include verification of which NAD^+^ biosynthesis pathway the patient’s tumor relies on. In this study, we demonstrated the importance of determining which pathway the tumor depends on for producing NAD^+^ by testing whether NMN or NA can reverse the drug effect, suggesting the importance of precision medicine in estimating KPT-9274 efficacy. However, it is currently challenging to rapidly and accurately determine which pathway is activated in a patient’s cancer cells, including methodology. Therefore, further validation is warranted to assess whether distinguishing NAMPT-dependency in patients prior to treatment for ovarian cancer can help improve the prognosis.

This study focuses primarily on cell line-based approaches using 3D-spheroids that mimic CSC enriched tumor masses floating intraperitoneally. While this model provides a variety of insights, in vivo studies are crucial for translating these findings into the clinical setting, particularly concerning drug efficacy and safety. Our findings in vitro studies suggest that further investigation of KPT-9274 in vivo is warranted. Overall, our preclinical data suggest that inhibiting NAMPT and PAK4 by KPT-9274 is an effective approach to overcome platinum resistance in ovarian cancers. These findings warrant further investigation to develop biomarkers to determine treatment efficacy of KPT-9274.

### Supplementary information

Supplementary items

## Data Availability

The RNA-seq datasets generated during this study are available in GEO (GSE251890). TCGA datasets are previously published and publicly available (https://www.cancer.gov/tcga). Other data (cell viability, caspase assays, seahorse assays) are available from the corresponding author on reasonable request.
