# Supplementary material for: Dual-inhibition of NAMPT and PAK4 induces anti-tumor effects in 3D-spheroids model of platinum-resistant ovarian cancer
Source: Cancer Gene Ther. 2024 Feb 29;31(5):721–35. doi: 10.1038/s41417-024-00748-w (PMC11101335; doi:10.1038/s41417-024-00748-w)
Supplement: Supplementary file 1 — Supplementary items [file 41417_2024_748_MOESM1_ESM.pdf]

Supplemental items

Supplementary Fig. 1

KPT-9274 is a promising compound for NAMPT-dependent 3D-spheroids, related to Fig. 1.

Supplementary Fig. 2

KPT-9274 causes cleaved caspase-3/7 from mitochondria, related to Fig. 3.

Supplementary Fig. 3

NAMPT positively modulates inflammation-related genes, while PAK4 up-regulates genes related to DNA repair and cell proliferation in ovarian cancer, related to Fig. 4.

Supplementary Fig. 4

KPT-9274 affected genes involved in Inflammation Signaling, related to Fig. 5.

Supplementary Fig. 5

KPT-9274 suppressed phosphorylation of S6 Ribosomal protein, AKT, and β-Catenin, related to Fig. 6.

Supplementary Fig. 6

In contrast to KPT-9274, FK-866 and GNE-617 suppressed NAD^+ production without affecting PAK4 localization, related to Fig. 6.

Supplementary Table 1

Top 300 DEGs when comparing tumors of patients with NAMPT high vs. low expression, related to Fig. 4.

Supplementary Table 2

Top 300 DEGs when comparing tumors of patients with PAK4 high vs. low expression, related to Fig. 4.

Supplementary Table 3

Top 300 DEGs when comparing Control to KPT-9274 treatment, related to Fig. 5.

Supplementary figure legends

Supplementary Fig. 1

KPT-9274 is a promising compound for NAMPT-dependent 3D-spheroids

(A) Chemical structure of KPT-9274

(B) TCGA analysis revealed that high expression of NAMPT in human cervical cancer and endometrial cancer significantly correlates with worse prognosis (Top left and top middle). In breast cancer, the correlation with worse prognosis is non-significant (Top right). Regarding PAK4, No differences were found in these cancers (Bottom), though high expression of PAK4 in human endometrioid cancer tend to have worse prognosis (Bottom middle).

(C) IC_{50} in cell lines that KPT-9274 treatment was effective in 3D-culture. Cell viability was assessed using XTT assay. (n = 4 independent experiments)

(D) Schematic diagram showing that NMN or NA is added into Stem Cell culture Media to evaluate the presence of NMN or NA rescue.

(E) Schematic diagram showing that NAMPT inhibitor treatment triggers cell death in NAD^+-dependent cell lines, despite NA presence in media. While the same treatment for cell lines that also synthesizes NAD^+ from NA lead to cell growth (NA rescue).

Graph data were presented as mean ± SEM with n = 4 per group.

Supplementary Fig. 2

KPT-9274 causes cleaved caspase-3/7 from mitochondria

(**A**) Left: Fluorescence analysis of ACI-98 spheroids after treatment with KPT-9274 at indicated doses. The spheroids were treated 3days after seeding cells. Time-dependent bright field and fluorescent overlay images of cleaved caspase-3/7 for the spheroids. Right: Quantification of Green Mean Intensity as a function of time (days) using IncuCyte^{TM} S3. (*n* = 4 independent experiments)

Graph data were presented as mean ± SEM with *n* = 4 per group.

Supplementary Fig. 3

NAMPT positively modulates inflammation-related genes, while PAK4 up-regulates genes related to DNA repair and cell proliferation in ovarian cancer

(A) Violin plots showing the relative RNA-seq signature of representative genes for analyzing the distribution of gene expression in patients with high (n=73) and low (n=73) NAMPT expression.

(B) GSEA in patients with NAMPT high relative to NAMPT low. (Left: Hallmark gene sets in MsigDB, Right: KEGG pathway DB)

(C) Violin plots showing the relative RNA-seq signature of representative genes for analyzing the distribution of gene expression in patients with PAK4 high (n=73) and PAK4 low (n=73).

(D) GSEA in patients with PAK4 high relative to PAK4 low. (Left: Hallmark gene sets in MsigDB, Right: KEGG pathway DB)

Supplementary Fig. 4

KPT-9274 affected genes involved in Inflammation Signaling

(A) Schematic of Interferon pathway showing increased (pink or orange) or decreased (green or blue) genes in 3D-cultured CP80 spheroids treated KPT-9274 1 000 nM for 24 h.

(B) Normalized gene expression levels associated with Interferon Signaling in Control and KPT-9274 treatment. (*n* = 4 independent experiments)

Graph data were presented as mean ± SEM with *n* = 4 per group.

Supplementary Fig. 5

KPT-9274 suppressed phosphorylation of S6 Ribosomal protein, AKT, and β-Catenin

(A) and **(B)** Immunoblotting using whole cell lysates for assessing the activity of DNA repair, Serine/threonine protein kinase, mTORC1, mTORC2, and Wnt/β-Catenin signaling in 3D-cultured **(A)** A2780 and **(B)** ACI-98 cells with KPT-9274 treatment at indicated doses. PAR for NAD^+-mediated DNA repair, PAK4 for Serine/threonine protein kinase, RAPTOR and the phosphorylation of S6 (p-S6) at S235/236 for mTORC1, the phosphorylation of AKT (p-AKT) at S473 for mTORC2, and the phosphorylation of β-Catenin (p-β-Catenin) at S675 for Wnt/β-Catenin signaling. Total S6, AKT, β-Catenin, and GAPDH are shown as controls.

Supplementary Fig. 6

In contrast to KPT-9274, FK-866 and GNE-617 suppressed NAD^+ production without affecting PAK4 localization.

(A) Change in total NAD levels in 3D-cultured CP80 with Control, FK-866 2 nM, and 4 nM. (*n* = 4 independent experiments)

(B) Immunoblotting for assessing the activity of NAMPT, Serine/threonine protein kinase, mTORC1, mTORC2, and Wnt/β-Catenin signaling in 3D-cultured CP80 cell lysates with Control, FK-866 2 nM, and 4 nM. The phosphorylation of S6 (p-S6) at S235/236 for mTORC1, the phosphorylation of AKT (p-AKT) at S473 for mTORC2, and the phosphorylation of β-Catenin (p-

β-Catenin) at S675 for Wnt/β-Catenin signaling. Total S6, AKT, β-Catenin, GAPDH, and LaminB1 were shown as controls. (Left: cytoplasm lysate, Right: nuclear lysate)

(C) Change in total NAD levels in 3D-cultured CP80 with Control, GNE-617 4 nM, and 8 nM. (*n* = 3 independent experiments)

(D) Immunoblotting for assessing the activity of NAMPT, mTORC1, mTORC2, and Wnt/β-Catenin signaling in 3D-cultured CP80 cell lysates with Control, GNE-617 4 nM, and 8 nM. The evaluated proteins are the same as those described in B.

Graph data were presented as mean ± SEM with *n* = 3 or 4 per group.

Supplementary Fig. 1

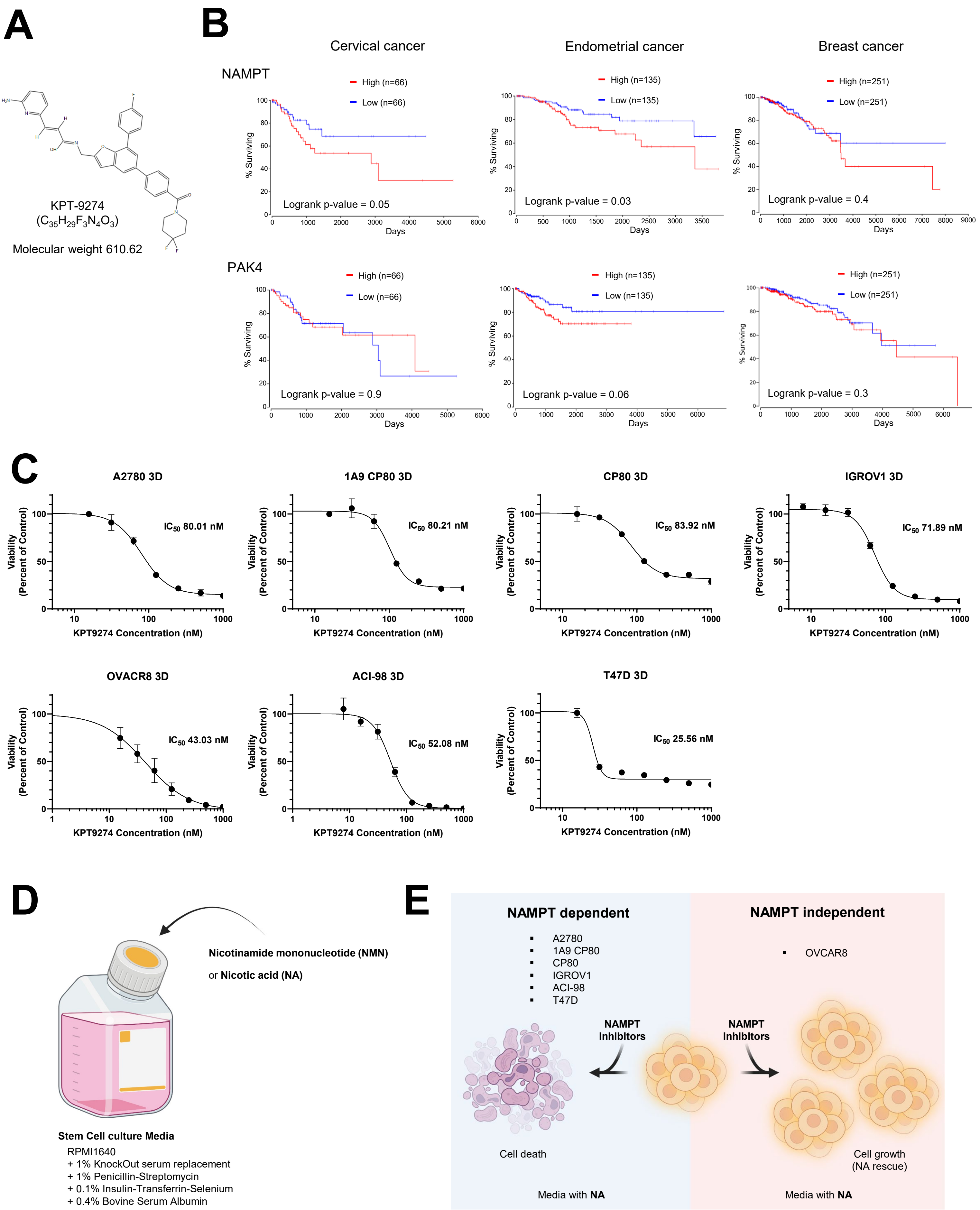

Supplementary Fig. 2

A

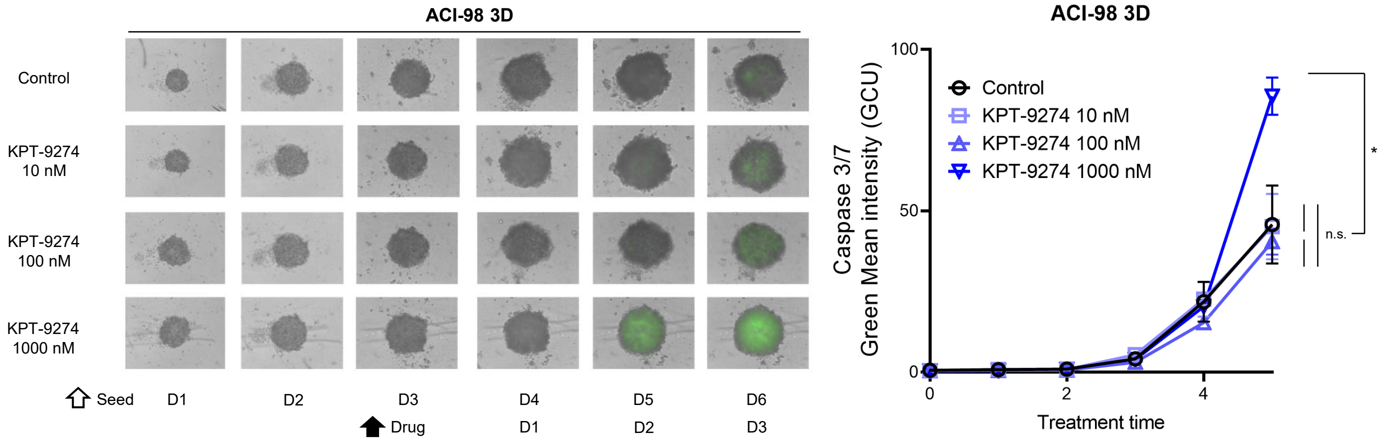

Supplementary Fig. 3

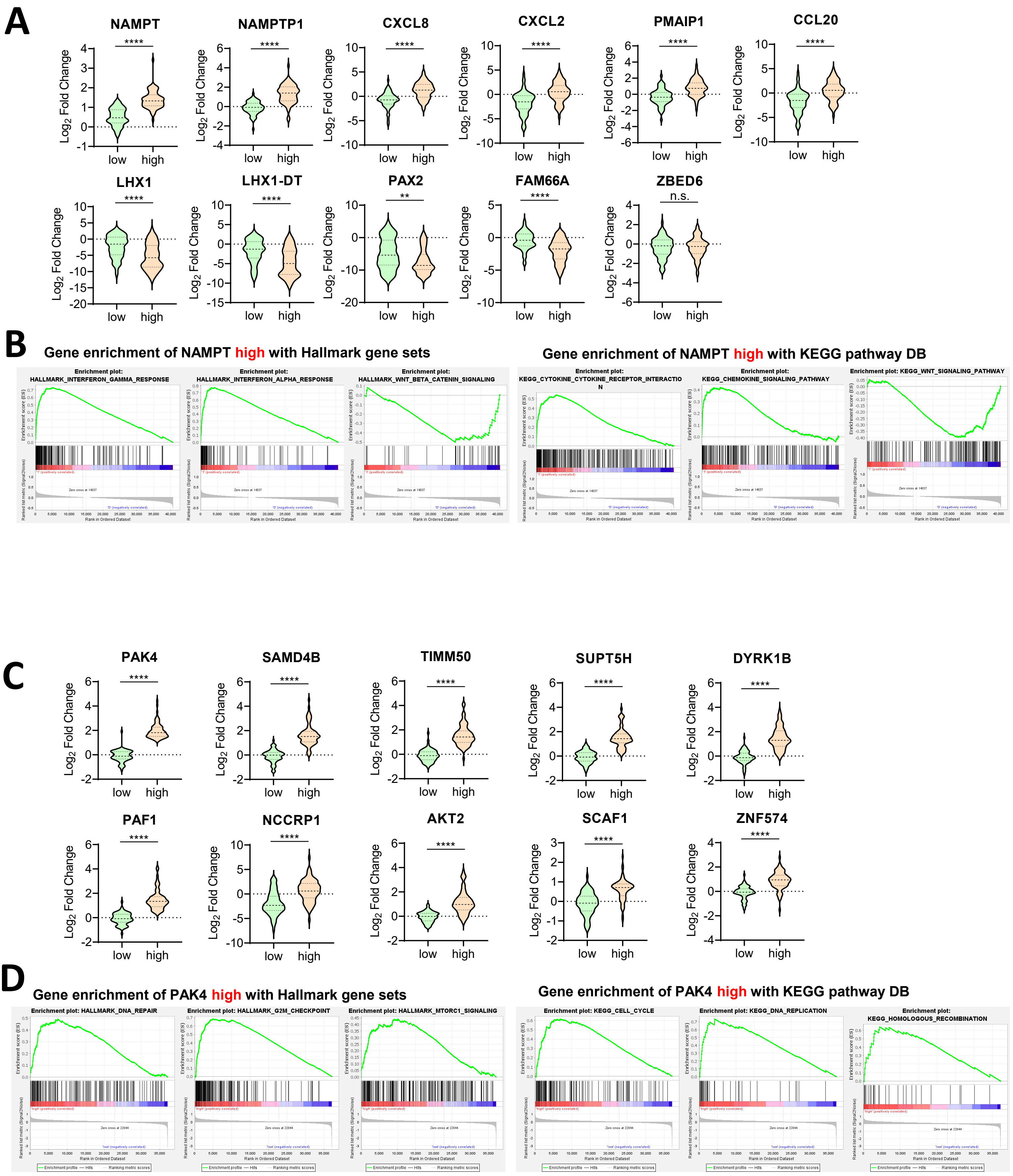

Supplementary Fig. 4

A

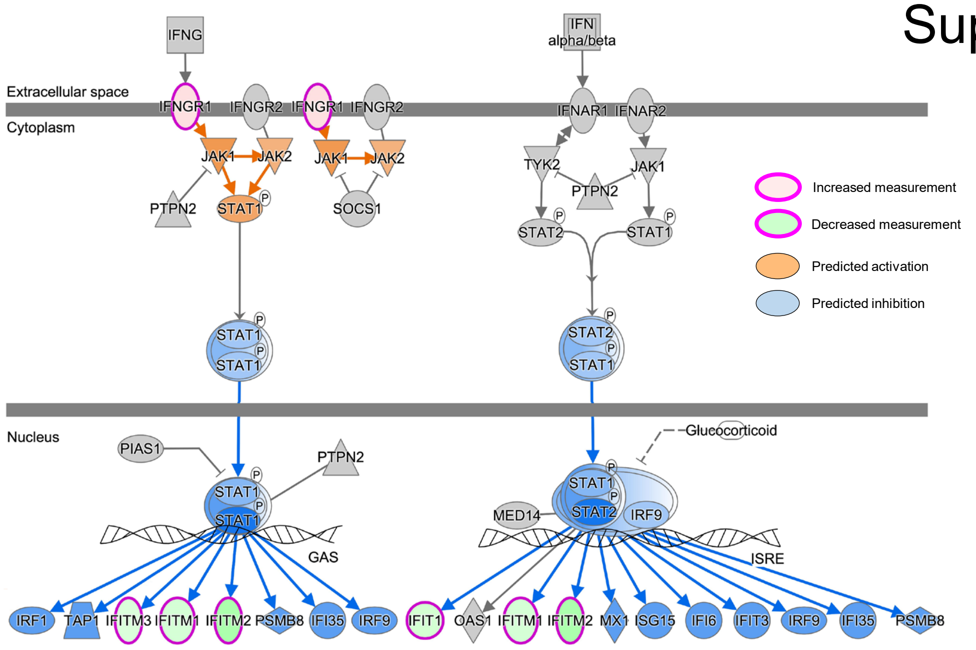

B

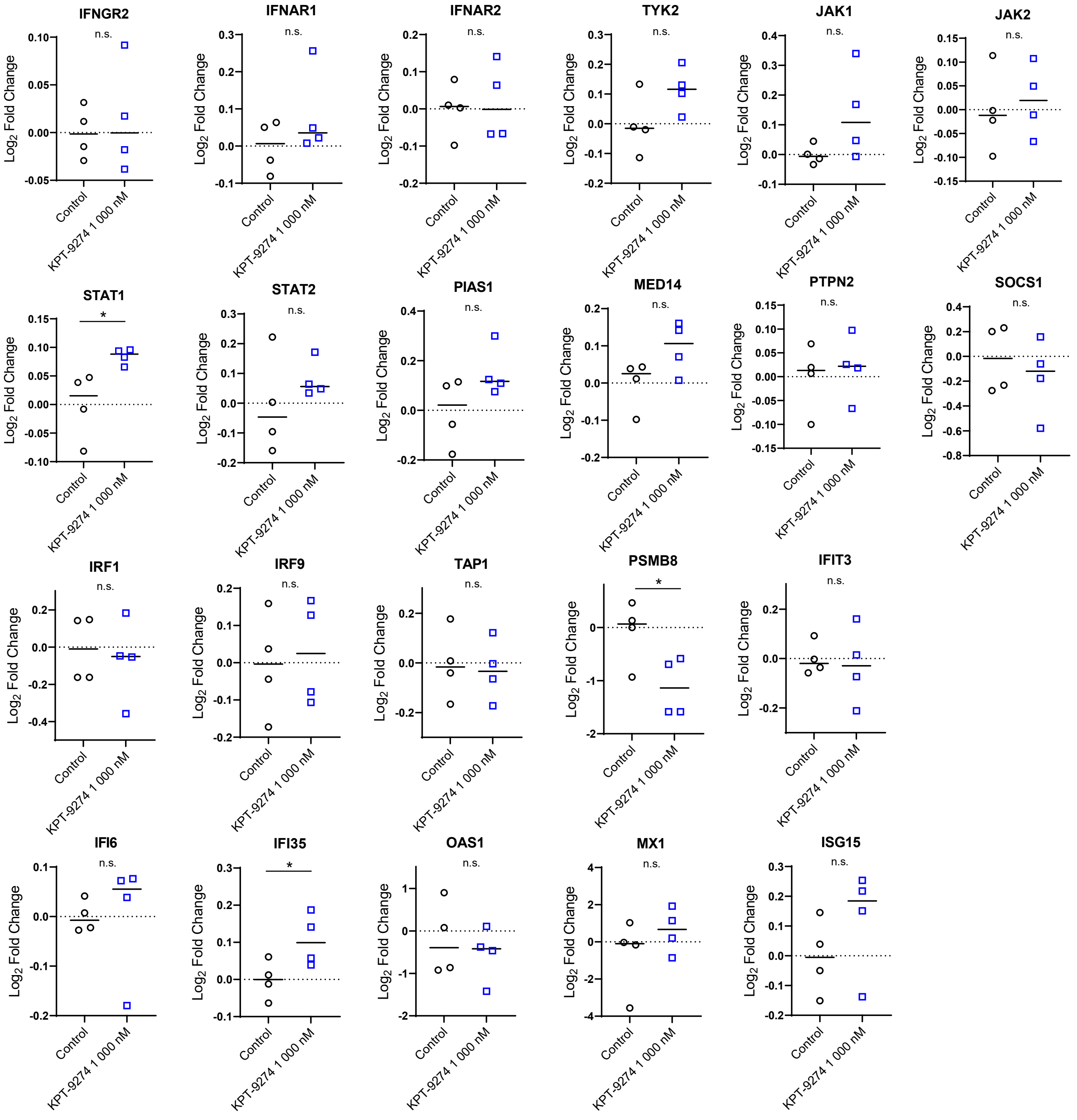

Supplementary Fig. 5

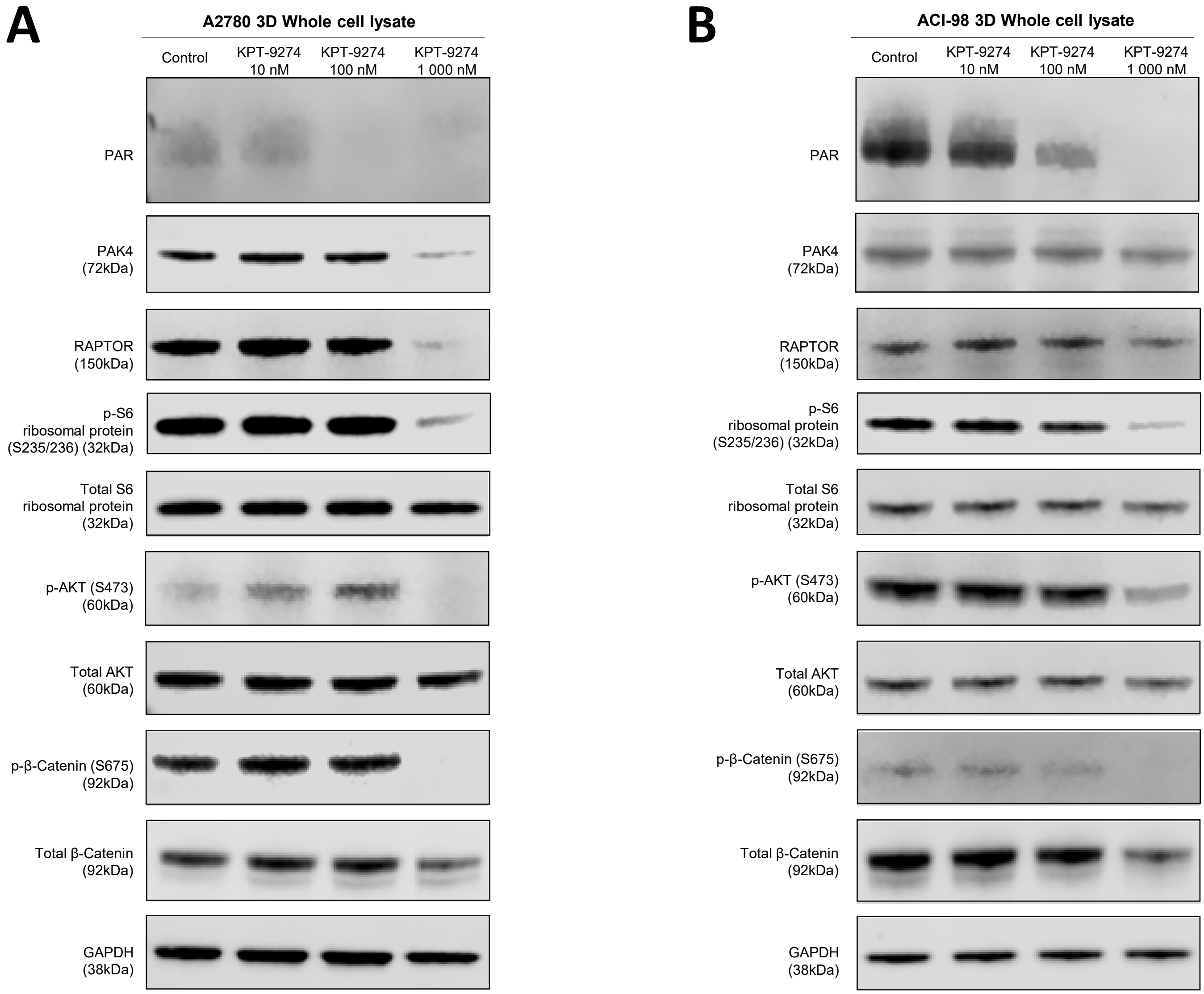

Supplementary Fig. 6

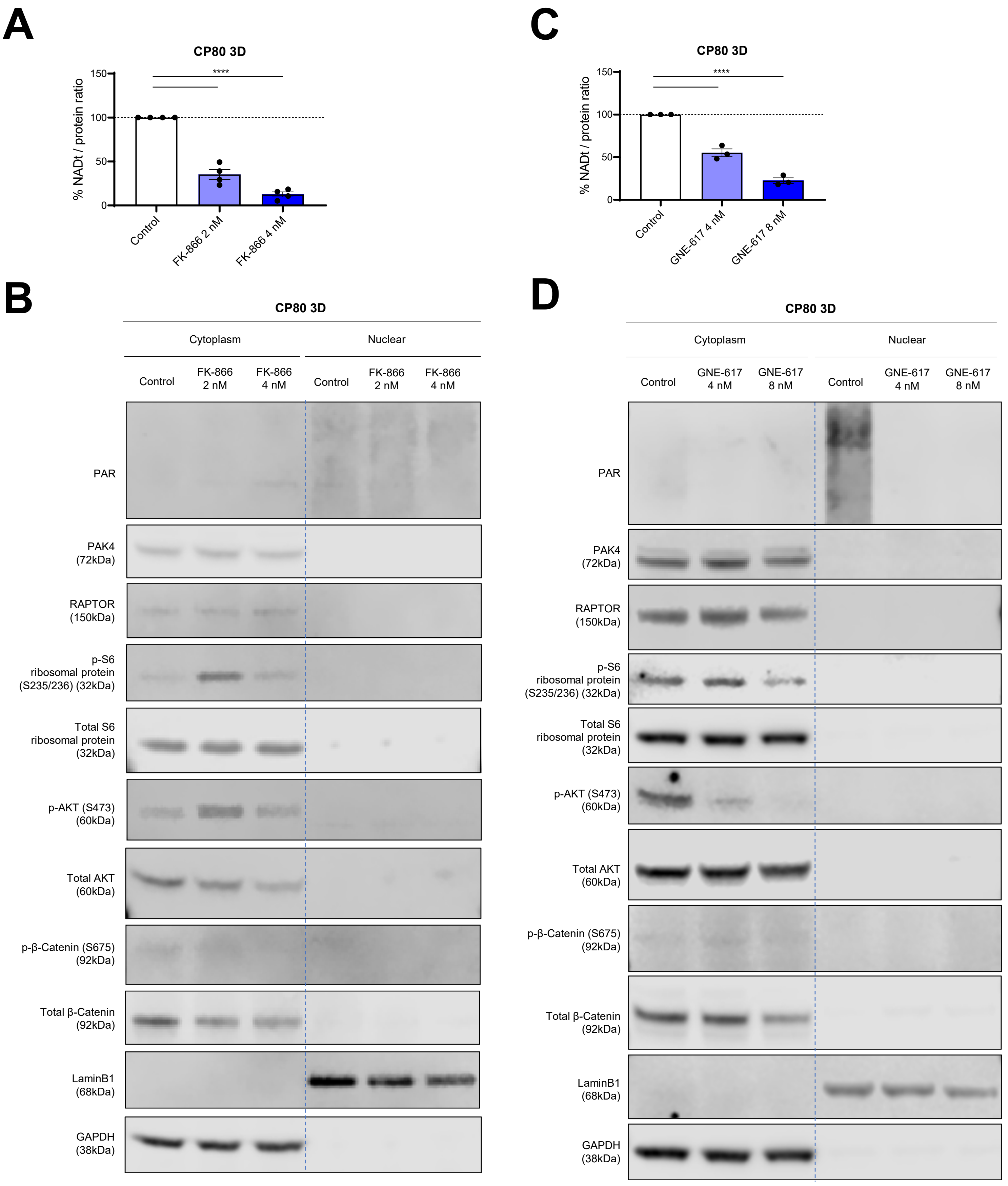

Supplementary Table 1

Rank	Gene name	p-value	Rank	Gene name	p-value	Rank	Gene name	p-value
1	NAMPT	7.5E-43	101	CACNA2D2	2.9E-05	201	PACRG	1.1E-04
2	NAMPTP1	7.2E-18	102	LINC-PINT	3.0E-05	202	MIR4753	1.1E-04
3	ARMC10	1.4E-12	103	FGD5P1	3.0E-05	203	ARFIP2	1.2E-04
4	CAPZA2	1.1E-11	104	CCL7	3.1E-05	204	PCDHB6	1.2E-04
5	CXCL8	2.1E-10	105	FAM166A	3.1E-05	205	MSMO1	1.2E-04
6	CCDC71L	2.5E-10	106	ITM2C	3.2E-05	206	TC2N	1.2E-04
7	NCOA7	1.2E-09	107	NAXD	3.3E-05	207	TSNAXIP1	1.2E-04
8	PMAIP1	2.4E-09	108	KATNB1	3.5E-05	208	GSTA4	1.2E-04
9	SYPL1	2.4E-09	109	CYP11A1	3.5E-05	209	LGI3	1.2E-04
10	PNPLA8	4.4E-09	110	RSAD1	3.6E-05	210	R3HCC1	1.3E-04
11	CXCL2	2.1E-08	111	CAMK2B	3.8E-05	211	PLEKHB1	1.3E-04
12	CEBPD	2.2E-08	112	PBXIP1	3.9E-05	212	FAM66E	1.3E-04
13	CCL20	4.5E-08	113	TNFSF10	3.9E-05	213	LRRC46	1.3E-04
14	ZBED6	5.7E-08	114	DNAJC2	4.0E-05	214	C17orf97	1.3E-04
15	FAM66A	1.3E-07	115	PTOV1	4.0E-05	215	DOK1	1.3E-04
16	PNMA8B	1.7E-07	116	PMPCB	4.1E-05	216	AC123768.2	1.3E-04
17	PYCR2	2.4E-07	117	SARDH	4.1E-05	217	FAS	1.4E-04
18	PSMC2	2.8E-07	118	FCAR	4.1E-05	218	KCNRG	1.4E-04
19	SOD2	2.9E-07	119	KCNH3	4.2E-05	219	CPA4	1.4E-04
20	STEAP1	5.0E-07	120	CXCL6	4.2E-05	220	CSTF2T	1.4E-04
21	NHLRC4	5.3E-07	121	MYH15	4.2E-05	221	P2RX6	1.4E-04
22	IL1RN	8.1E-07	122	VPS50	4.2E-05	222	NBEA	1.4E-04
23	RINT1	8.6E-07	123	RGL3	4.2E-05	223	TMEM151B	1.4E-04
24	THAP5	8.9E-07	124	GTPBP10	4.3E-05	224	CAMK1G	1.4E-04
25	AL139011.1	9.5E-07	125	PLSCR1	4.4E-05	225	SRCIN1	1.4E-04
26	IFT27	1.0E-06	126	OSMR	4.4E-05	226	AC005670.3	1.4E-04
27	NNMT	1.3E-06	127	SEPTIN9	4.4E-05	227	NIT1	1.5E-04
28	SLC11A1	1.4E-06	128	IL6	4.4E-05	228	AC110611.2	1.5E-04
29	PUS7	1.5E-06	129	PELATON	4.5E-05	229	PI3	1.5E-04
30	CXCL3	1.7E-06	130	GP5	4.5E-05	230	PLAUR	1.5E-04
31	SCRN2	2.0E-06	131	MTX1	4.6E-05	231	SAMSN1	1.6E-04
32	IFNGR1	2.3E-06	132	TMEM165	4.7E-05	232	BFSP1	1.6E-04
33	MKS1	2.3E-06	133	AC138904.3	4.7E-05	233	LAMA5-AS1	1.6E-04

34	DNAJB9	2.8E-06	134	DDIT4L	4.8E-05	234	IFRD1	1.6E-04
35	ZNF277	2.8E-06	135	CBLL1	4.9E-05	235	LYSMD4	1.6E-04
36	ENO4	2.9E-06	136	CXCL1	5.0E-05	236	ZNF688	1.6E-04
37	ZC3H12A	3.2E-06	137	ZNF853	5.1E-05	237	H3C9P	1.6E-04
38	LHX1-DT	3.4E-06	138	S100A12	5.1E-05	238	ESS2	1.6E-04
39	DTX3	3.4E-06	139	KLHDC9	5.1E-05	239	TXNRD2	1.7E-04
40	RHEB	4.2E-06	140	EFR3B	5.2E-05	240	GALNT2	1.7E-04
41	ZDHHC1	4.4E-06	141	COG5	5.4E-05	241	RADIL	1.7E-04
42	MACROH2A2	4.4E-06	142	SMIM6	5.4E-05	242	ANKRD45	1.7E-04
43	ARPC1A	4.5E-06	143	ANKRD54	5.5E-05	243	STPG1	1.7E-04
44	KDM8	4.8E-06	144	DBH	5.5E-05	244	NUP54	1.7E-04
45	LHX1	4.8E-06	145	VAX2	5.8E-05	245	DZIP3	1.7E-04
46	FIGNL2	5.5E-06	146	LGSN	6.0E-05	246	IFIT2	1.7E-04
47	PPOX	6.3E-06	147	LINC01094	6.1E-05	247	DRC3	1.7E-04
48	POLM	6.3E-06	148	SMIM30	6.2E-05	248	AC004839.1	1.8E-04
49	NOXA1	7.1E-06	149	RSBN1L	6.5E-05	249	FAM166C	1.8E-04
50	AL513534.2	7.2E-06	150	CHADL	6.6E-05	250	COL28A1	1.8E-04
51	RNF187	7.4E-06	151	DZANK1	6.6E-05	251	TMEM89	1.8E-04
52	MCEMP1	7.6E-06	152	RBKS	6.6E-05	252	SPPL2B	1.8E-04
53	AC008760.2	8.1E-06	153	SOCS3	6.7E-05	253	SALL2	1.8E-04
54	BCL2A1	8.1E-06	154	CCDC102A	6.9E-05	254	SPP1	1.8E-04
55	CXCL10	8.1E-06	155	TNFSF13B	6.9E-05	255	MCUB	1.8E-04
56	MARCHF10	9.9E-06	156	TNNI1	6.9E-05	256	ACTR3	1.8E-04
57	PRCC	9.9E-06	157	AHR	7.1E-05	257	VNN1	1.9E-04
58	SPEF1	1.0E-05	158	CYTH2	7.3E-05	258	FIGNL2-DT	1.9E-04
59	RNASE2	1.0E-05	159	CREB3L4	7.4E-05	259	CUEDC1	1.9E-04
60	IER3IP1	1.0E-05	160	NES	7.4E-05	260	LRRC27	1.9E-04
61	FAM3C	1.1E-05	161	PYM1	7.5E-05	261	BET1	2.0E-04
62	NCK1	1.1E-05	162	WASHC1	7.5E-05	262	FGF17	2.0E-04
63	PPIAP14	1.1E-05	163	OSTM1	7.7E-05	263	SF3A2	2.0E-04
64	SRGN	1.1E-05	164	TTLL1-AS1	7.8E-05	264	KCNN3	2.0E-04
65	IMMP2L	1.2E-05	165	NMI	8.1E-05	265	GBP1	2.0E-04
66	PRKAG2	1.2E-05	166	RGS1	8.1E-05	266	SDS	2.0E-04
67	TSSC4	1.3E-05	167	RAB37	8.1E-05	267	MIR4648	2.0E-04

68	SH3BP5L	1.3E-05	168	PRR29-AS1	8.2E-05	268	FAM3C2P	2.0E-04
69	CIRBP	1.3E-05	169	GSDMC	8.3E-05	269	ACSM1	2.1E-04
70	ZSCAN2	1.3E-05	170	PDZK1IP1	8.3E-05	270	KLHL22	2.1E-04
71	CCDC151	1.4E-05	171	OTUB1	8.4E-05	271	RASA4CP	2.1E-04
72	KAT14	1.5E-05	172	PDK2	8.4E-05	272	ERO1A	2.1E-04
73	MYOSLID	1.6E-05	173	MPRIPP1	8.5E-05	273	TRIB1	2.1E-04
74	ME2	1.6E-05	174	OCM	8.6E-05	274	ADAM33	2.1E-04
75	CD69	1.7E-05	175	BIRC3	8.7E-05	275	GTF3C1	2.1E-04
76	IGSF8	1.8E-05	176	PRR29	8.9E-05	276	TCTE1	2.2E-04
77	OSM	1.8E-05	177	CDK20	9.0E-05	277	NFKBIA	2.2E-04
78	NFKBIZ	1.8E-05	178	ZFHX2	9.0E-05	278	SLC4A8	2.2E-04
79	DBH-AS1	1.9E-05	179	ATF3	9.0E-05	279	RAB36	2.2E-04
80	DCAKD	1.9E-05	180	AC016705.2	9.0E-05	280	SBDS	2.2E-04
81	TREM1	1.9E-05	181	CCDC74B	9.1E-05	281	ABCD4	2.2E-04
82	ZNF800	2.0E-05	182	IFI16	9.2E-05	282	MAGEF1	2.3E-04
83	DCK	2.0E-05	183	MICB	9.2E-05	283	HAGH	2.3E-04
84	HEXIM2	2.0E-05	184	AC067852.2	9.3E-05	284	NCKAP5L	2.3E-04
85	AC079336.6	2.0E-05	185	XXYLT1	9.4E-05	285	EMILIN3	2.3E-04
86	RSPH14	2.1E-05	186	SLC26A4	9.5E-05	286	RECQL5	2.3E-04
87	CACFD1	2.2E-05	187	OGFOD3	9.5E-05	287	GP1BA	2.4E-04
88	BRI3	2.2E-05	188	AC012354.1	9.7E-05	288	MAPRE3	2.4E-04
89	AC016831.6	2.2E-05	189	CSTA	9.8E-05	289	PPIAP74	2.4E-04
90	SMARCB1	2.2E-05	190	BCAS3	9.9E-05	290	TSPAN7	2.4E-04
91	OLR1	2.3E-05	191	CXCL11	1.0E-04	291	C1orf226	2.4E-04
92	EREG	2.4E-05	192	EVA1C	1.0E-04	292	UGCG	2.4E-04
93	RBM8A	2.4E-05	193	USH1C	1.0E-04	293	SLC17A8	2.4E-04
94	SCAMP5	2.4E-05	194	USP21	1.0E-04	294	COL18A1	2.5E-04
95	KLHL25	2.4E-05	195	CARD10	1.0E-04	295	LACTB	2.5E-04
96	B4GALT3	2.5E-05	196	DMTN	1.0E-04	296	LGALS3	2.6E-04
97	ORC5	2.5E-05	197	SEMA6C	1.0E-04	297	AC004066.1	2.6E-04
98	WDR18	2.6E-05	198	FPR2	1.1E-04	298	NCOA5	2.6E-04
99	DLD	2.8E-05	199	AC015813.8	1.1E-04	299	LIPN	2.6E-04
100	ADAMTS7P1	2.8E-05	200	STK17B	1.1E-04	300	ENTPD8	2.6E-04

Supplementary Table 2

Rank	Gene name	p-value	Rank	Gene name	p-value	Rank	Gene name	p-value
1	PAK4	1.1E-42	101	ZNF566	2.9E-10	201	ZNF793-AS1	7.9E-08
2	SAMD4B	6.9E-27	102	ZNF570	3.0E-10	202	MKI67	8.5E-08
3	TIMM50	1.8E-26	103	CATSPERG	3.6E-10	203	GRIK5	8.5E-08
4	SUPT5H	2.6E-26	104	TIMELESS	4.1E-10	204	AL031658.1	8.5E-08
5	PAF1	8.1E-26	105	OPA3	4.5E-10	205	CDC25A	8.6E-08
6	ECH1	1.0E-23	106	AC016590.2	5.2E-10	206	AL353807.5	8.7E-08
7	EID2	1.1E-23	107	CLPTM1	6.4E-10	207	NANOS1	9.4E-08
8	DYRK1B	1.7E-23	108	ZFP14	8.7E-10	208	TLE1P1	9.7E-08
9	MED29	4.4E-23	109	ZNF383	8.7E-10	209	CCDC8	1.0E-07
10	SARS2	5.7E-23	110	RPS16	9.6E-10	210	ABL1	1.0E-07
11	ACTN4	1.9E-22	111	ZNF45	9.9E-10	211	PRMT1	1.0E-07
12	PSMC4	6.5E-21	112	ITPKC	1.0E-09	212	ZNF222	1.0E-07
13	YIF1B	8.3E-21	113	INA	1.1E-09	213	ZNF461	1.1E-07
14	PLEKHG2	4.5E-20	114	PAFAH1B3	1.1E-09	214	XKR7	1.1E-07
15	SIPA1L3	1.1E-19	115	ANKRD27	1.2E-09	215	LMNB2	1.1E-07
16	NFKBIB	1.7E-19	116	ZNF573	1.2E-09	216	SIX5	1.1E-07
17	FBL	1.8E-19	117	CADM4	1.3E-09	217	MSI1	1.1E-07
18	PSMD8	3.3E-18	118	MAP4K1	1.3E-09	218	SERTAD3	1.1E-07
19	HNRNPL	4.6E-18	119	ZNF829	1.4E-09	219	TMEFF1	1.1E-07
20	SPINT2	1.3E-17	120	MARK4	1.4E-09	220	BICRA	1.2E-07
21	FBXO27	2.7E-17	121	DLL3	1.5E-09	221	ZNF565	1.2E-07
22	AC011455.8	5.1E-17	122	PRX	1.6E-09	222	LRFN3	1.2E-07
23	MRPS12	5.9E-17	123	DMWD	1.8E-09	223	TICRR	1.2E-07
24	ZNF607	5.9E-17	124	CENPO	1.9E-09	224	CNOT3	1.2E-07
25	ERVK9-11	6.6E-17	125	NCCRP1	2.1E-09	225	MLEC	1.3E-07
26	ZNF780A	2.7E-16	126	CCER2	2.2E-09	226	U2AF1L4	1.3E-07
27	AKT2	4.4E-16	127	AC104534.1	2.2E-09	227	RAD51AP1	1.4E-07
28	ZFP30	1.0E-15	128	LSM14A	2.3E-09	228	ZNF473	1.4E-07
29	GSK3A	1.3E-15	129	UBA2	2.4E-09	229	AC016590.3	1.5E-07
30	HNRNPUL1	2.6E-15	130	APLP1	2.5E-09	230	ZNF283	1.5E-07
31	ACTN4P1	3.6E-15	131	TRIM28	2.7E-09	231	SMTNL2	1.5E-07
32	ZNF875	4.7E-15	132	AC011465.1	2.7E-09	232	USP44	1.5E-07
33	LRFN1	8.8E-15	133	IRGQ	2.8E-09	233	ETV2	1.5E-07

34	SHKBP1	9.5E-15	134	ZNF569	3.4E-09	234	RBM14	1.6E-07
35	C19orf47	1.0E-14	135	TBCB	4.1E-09	235	MYBL2	1.6E-07
36	ZNF574	1.1E-14	136	ZNF529	4.2E-09	236	HLA-DMA	1.6E-07
37	AC005393.1	2.1E-14	137	CAPNS1	4.5E-09	237	TMEM147	1.6E-07
38	EID2B	3.3E-14	138	FAAP24	4.8E-09	238	SMC2	1.7E-07
39	FBXO17	4.7E-14	139	HIPK4	5.0E-09	239	FANCC	1.8E-07
40	ZNF780B	5.5E-14	140	ZNF507	5.1E-09	240	CIT	1.8E-07
41	SIRT2	6.0E-14	141	ZNF428	5.3E-09	241	ERCC2	1.9E-07
42	ZNF850	7.9E-14	142	PPP5C	5.3E-09	242	ATXN7L3B	1.9E-07
43	RYR1	8.4E-14	143	POLD1	5.6E-09	243	CKAP4	1.9E-07
44	PROSER3	1.8E-13	144	CEP89	5.7E-09	244	GAREM2	1.9E-07
45	ZNF345	2.4E-13	145	AC012306.3	6.4E-09	245	BRCA2	1.9E-07
46	CCDC97	3.0E-13	146	GPATCH1	9.1E-09	246	ODF2	2.1E-07
47	EIF3K	3.9E-13	147	CENPF	9.5E-09	247	TPX2	2.1E-07
48	ZNF260	5.8E-13	148	PRR19	1.0E-08	248	PVR	2.2E-07
49	WDR62	6.6E-13	149	PLEKHG6	1.1E-08	249	FOXM1	2.2E-07
50	ZNF585B	7.6E-13	150	SMG9	1.1E-08	250	DHX34	2.3E-07
51	HAUS5	8.8E-13	151	AC022144.1	1.2E-08	251	ZNF382	2.4E-07
52	AC008982.2	8.8E-13	152	PSENEN	1.2E-08	252	ZNF618	2.4E-07
53	TOMM40	9.1E-13	153	NCAPD2	1.2E-08	253	HLA-DRB1	2.5E-07
54	NECTIN2	1.1E-12	154	ZNF235	1.3E-08	254	EFR3B	2.5E-07
55	ZFP82	1.1E-12	155	ODC1	1.4E-08	255	ZNF227	2.6E-07
56	ZNF793	1.5E-12	156	AC011445.1	1.4E-08	256	H2AZ2	2.6E-07
57	LGALS7B	2.4E-12	157	CALM3	1.5E-08	257	AC092295.2	2.7E-07
58	ZNF567	2.7E-12	158	SPRED3	1.5E-08	258	C1GALT1C1L	2.7E-07
59	GGN	2.8E-12	159	ZBTB39	1.5E-08	259	PTOV1	2.8E-07
60	LGALS4	3.3E-12	160	FIZ1	1.6E-08	260	CCNDBP1	2.8E-07
61	ZNF180	3.6E-12	161	CEP78	1.7E-08	261	BCL2L12	3.1E-07
62	ZNF585A	3.7E-12	162	NCAPH	1.8E-08	262	WBP11	3.1E-07
63	ZNF568	4.1E-12	163	FKRP	1.8E-08	263	FBRSL1	3.4E-07
64	RBM42	6.1E-12	164	ARHGAP35	1.9E-08	264	HLA-DPB1	3.7E-07
65	ZNF420	7.6E-12	165	MED25	1.9E-08	265	KCNJ14	3.8E-07
66	MAP3K10	8.0E-12	166	DBF4B	2.0E-08	266	ZNF444	4.0E-07
67	LINC00665	1.2E-11	167	CENPBD1P1	2.0E-08	267	STRBP	4.1E-07

68	MEGF8	1.3E-11	168	DUSP23	2.0E-08	268	AC002398.1	4.1E-07
69	ZNF571	1.4E-11	169	KIRREL2	2.1E-08	269	IL15RA	4.2E-07
70	ZNF526	1.6E-11	170	NUMBL	2.2E-08	270	AC012073.1	4.2E-07
71	AC011479.3	1.6E-11	171	SNRPA	2.4E-08	271	ASPM	4.2E-07
72	DPF1	1.6E-11	172	NUP62	2.5E-08	272	PRR12	4.3E-07
73	STRN4	1.9E-11	173	RUVBL2	3.3E-08	273	BEND3	4.3E-07
74	CIC	2.0E-11	174	ZNF230	3.3E-08	274	HLA-DRA	4.4E-07
75	SAE1	2.0E-11	175	AC118344.1	3.4E-08	275	AC011479.2	4.5E-07
76	KMT2B	2.0E-11	176	RACGAP1	3.4E-08	276	VRK3	4.5E-07
77	SCAF1	2.1E-11	177	LIPE	3.6E-08	277	SYMPK	4.7E-07
78	CAPN12	2.3E-11	178	AC093227.1	3.7E-08	278	TEX15	4.8E-07
79	ERF	3.4E-11	179	ATXN7L3	3.8E-08	279	USH1G	4.8E-07
80	ZNF146	3.9E-11	180	KMT5C	4.0E-08	280	ZNF114	4.9E-07
81	PLD3	4.1E-11	181	CDC6	4.0E-08	281	AC016727.1	4.9E-07
82	RN7SL566P	4.5E-11	182	ZNF576	4.2E-08	282	TMPO	4.9E-07
83	COQ8B	6.6E-11	183	POLR1G	4.3E-08	283	Y_RNA	5.0E-07
84	FBXO46	6.6E-11	184	CEBPG	4.3E-08	284	ECT2	5.1E-07
85	FAM98C	6.7E-11	185	KRT8	4.4E-08	285	HNRNPLP1	5.1E-07
86	DEDD2	7.1E-11	186	NEMP1	4.5E-08	286	SBK1	5.1E-07
87	C19orf54	7.9E-11	187	ESPL1	4.6E-08	287	GFRA3	5.2E-07
88	ZNF527	1.1E-10	188	THAP8	4.7E-08	288	ARHGAP33	5.4E-07
89	ZNF790	1.1E-10	189	PNMA8A	4.9E-08	289	NCBP1	5.5E-07
90	PPP1R37	1.3E-10	190	BLM	5.1E-08	290	GTSE1	5.6E-07
91	XRCC1	1.3E-10	191	TOP2A	5.1E-08	291	CLASRP	5.7E-07
92	SPHK2	1.4E-10	192	PSMB8	5.6E-08	292	SP2	5.7E-07
93	NPAS1	1.6E-10	193	SNRNP200	5.7E-08	293	PHLDB3	5.8E-07
94	IRF2BP1	1.6E-10	194	UPK1A	5.8E-08	294	E2F7	5.9E-07
95	ZNF546	1.6E-10	195	PRKDC	5.8E-08	295	TFG	6.0E-07
96	DMAC2	1.7E-10	196	BRIP1	5.8E-08	296	INCENP	6.1E-07
97	AC008649.2	1.8E-10	197	POLE	6.0E-08	297	CCDC150	6.1E-07
98	ZSCAN5A	2.3E-10	198	MCM4	6.8E-08	298	IFNL4	6.1E-07
99	LIG1	2.5E-10	199	HROB	7.6E-08	299	KDM2B	6.2E-07
100	AC007842.1	2.9E-10	200	KIF18B	7.7E-08	300	GNAQP1	6.2E-07

Supplementary Table 3

Rank	Gene name	p-value	Rank	Gene name	p-value	Rank	Gene name	p-value
1	CA14	9.7E-07	101	SRP14	3.4E-04	201	EBP	8.9E-04
2	NLGN3	1.1E-06	102	EGR1	3.7E-04	202	DTX2	9.0E-04
3	SCARA5	3.6E-06	103	FRY	3.7E-04	203	TRPC4AP	9.0E-04
4	HDGF	3.6E-06	104	CLIC4	3.7E-04	204	TMEM106C	9.1E-04
5	NQO1	4.8E-06	105	COL1A1	3.7E-04	205	SOCS2	9.3E-04
6	HMGA2	6.2E-06	106	CAVIN1	3.7E-04	206	TMSB4X	9.3E-04
7	ERP27	1.1E-05	107	RANBP1	3.8E-04	207	PIK3C2G	9.5E-04
8	HSD17B7	1.2E-05	108	UBE2L6	3.8E-04	208	GGCX	9.6E-04
9	PPP2R5B	1.3E-05	109	ERRFI1	3.8E-04	209	PPM1M	9.7E-04
10	MYOF	1.3E-05	110	COX19	3.8E-04	210	ZNF275	9.7E-04
11	PYM1	1.6E-05	111	CC2D1B	3.8E-04	211	TMEM127	9.8E-04
12	CDC42EP4	2.4E-05	112	SYT11	3.8E-04	212	GTF3C1	9.8E-04
13	ACTA2	2.4E-05	113	FIBIN	3.9E-04	213	AARD	9.8E-04
14	NQO2	2.8E-05	114	DKK1	3.9E-04	214	FKBP5	9.8E-04
15	YIPF6	2.9E-05	115	GYG1	3.9E-04	215	MAP1LC3A	9.9E-04
16	ATXN2	3.0E-05	116	CCDC144CP	4.0E-04	216	S100A11	9.9E-04
17	PTMA	3.6E-05	117	LIMA1	4.0E-04	217	FBF1	1.0E-03
18	SLC30A8	3.7E-05	118	GNG11	4.0E-04	218	TOB1	1.0E-03
19	SCN9A	3.7E-05	119	RPL9P25	4.1E-04	219	PHKA1P1	1.0E-03
20	ZBTB2	4.2E-05	120	FNDC5	4.1E-04	220	UNC5B	1.0E-03
21	PBX3	4.3E-05	121	IFIT1	4.1E-04	221	TMEM115	1.0E-03
22	BST2	4.5E-05	122	CHKB-DT	4.1E-04	222	LAPTM4A	1.0E-03
23	MAP4K3-DT	4.8E-05	123	ARHGAP28	4.2E-04	223	MEOX1	1.1E-03
24	EDIL3	5.0E-05	124	IFNGR1	4.3E-04	224	SGTB	1.1E-03
25	ARRDC4	5.2E-05	125	COLEC11	4.3E-04	225	H6PD	1.1E-03
26	TOMM70	5.9E-05	126	TOR2A	4.4E-04	226	MMRN2	1.1E-03
27	SFRP1	5.9E-05	127	FAXDC2	4.5E-04	227	FAM9C	1.1E-03
28	ARF6	6.2E-05	128	PCAT14	4.7E-04	228	AC007842.1	1.1E-03
29	CDH10	6.3E-05	129	SLC29A1	4.7E-04	229	S100A4	1.1E-03
30	COL3A1	6.6E-05	130	AC106886.2	4.7E-04	230	GCNT1	1.1E-03
31	TUBB4B	6.7E-05	131	BRD2	4.8E-04	231	UBE2N	1.1E-03
32	H1F0	6.9E-05	132	ADGRE5	4.8E-04	232	NOLC1	1.1E-03
33	PLAU	7.5E-05	133	MYH11	4.8E-04	233	KAT2B	1.2E-03

34	VCL	7.6E-05	134	WDR37	5.0E-04	234	AC133785.1	1.2E-03
35	GNAI1	8.2E-05	135	DGKQ	5.1E-04	235	CXCR4	1.2E-03
36	UBE2J2	8.4E-05	136	ATF5	5.1E-04	236	A1BG-AS1	1.2E-03
37	CPED1	8.7E-05	137	MEST	5.1E-04	237	WDTC1	1.2E-03
38	STARD4	9.2E-05	138	NAIP	5.2E-04	238	ETFB	1.2E-03
39	HOXC6	9.9E-05	139	ZDHHC7	5.3E-04	239	IPO13	1.2E-03
40	TMEM119	1.0E-04	140	SORT1	5.4E-04	240	CCT8	1.2E-03
41	IGF2BP3	1.1E-04	141	IFITM1	5.5E-04	241	POC1B-GALNT4	1.2E-03
42	DNAJB1	1.1E-04	142	KLHL9	5.6E-04	242	CYFIP2	1.2E-03
43	MRPS34	1.2E-04	143	AC009269.5	5.6E-04	243	HID1	1.2E-03
44	RSU1	1.2E-04	144	CCDC28A	5.7E-04	244	MIR22HG	1.2E-03
45	ATP7A	1.2E-04	145	APOL2	5.7E-04	245	BCCIP	1.2E-03
46	USP11	1.2E-04	146	NAGK	5.7E-04	246	OLFM3	1.2E-03
47	PFDN1	1.3E-04	147	MYC	5.7E-04	247	PREX1	1.2E-03
48	TBR1	1.3E-04	148	PLPPR2	5.8E-04	248	IQCE	1.2E-03
49	ABCA5	1.3E-04	149	CD248	5.8E-04	249	DYNC1I2	1.3E-03
50	MAB21L2	1.4E-04	150	NXPH4	5.8E-04	250	PSRC1	1.3E-03
51	ZNF91	1.4E-04	151	HOXB9	6.0E-04	251	PANX2	1.3E-03
52	GTF3C3	1.4E-04	152	HIPK2	6.1E-04	252	ZMAT3	1.3E-03
53	ZNF888	1.4E-04	153	PGF	6.2E-04	253	SEC24C	1.3E-03
54	RALGPS1	1.5E-04	154	MEAF6	6.2E-04	254	MGAT4A	1.3E-03
55	XKR7	1.5E-04	155	GRIP2	6.2E-04	255	PTGES2	1.4E-03
56	TBC1D20	1.5E-04	156	DNAJC28	6.3E-04	256	CES4A	1.4E-03
57	PFKFB4	1.5E-04	157	PMP22	6.3E-04	257	DHRS1	1.4E-03
58	OGDH	1.5E-04	158	RARG	6.4E-04	258	TMEM8B	1.4E-03
59	CYB5R1	1.5E-04	159	DNAJB9	6.4E-04	259	IL17RA	1.4E-03
60	PINK1	1.5E-04	160	CCDC80	6.5E-04	260	LPGAT1	1.4E-03
61	FOSB	1.6E-04	161	BCOR	6.5E-04	261	FITM2	1.4E-03
62	TSHZ1	1.6E-04	162	CLIC1	6.5E-04	262	ITGB1BP1	1.4E-03
63	SPRY4	1.6E-04	163	HLA-DPA1	6.5E-04	263	SSBP3	1.4E-03
64	MSRB3	1.7E-04	164	KIAA1191	6.6E-04	264	ADI1	1.4E-03
65	HECA	1.8E-04	165	DKK2	6.7E-04	265	DUSP3	1.4E-03
66	SBDSP1	1.8E-04	166	TNFRSF10D	6.7E-04	266	TP53INP1	1.4E-03
67	TJAP1	1.8E-04	167	SLC38A2	6.7E-04	267	RCAN3AS	1.4E-03

68	S100A16	1.9E-04	168	RTL5	6.8E-04	268	LIN28A	1.4E-03
69	MXD1	1.9E-04	169	WBP2	6.9E-04	269	RHOB	1.4E-03
70	KREMEN1	2.0E-04	170	H3F3B	6.9E-04	270	EBI3	1.4E-03
71	FBXO21	2.1E-04	171	COL5A2	6.9E-04	271	AC019080.5	1.5E-03
72	ARMCX3	2.1E-04	172	CTDSP2	7.0E-04	272	R3HCC1	1.5E-03
73	ARMC7	2.1E-04	173	CCDC34	7.0E-04	273	IER5	1.5E-03
74	TUBA1A	2.1E-04	174	FBXO10	7.1E-04	274	NEBL	1.5E-03
75	ATF4	2.1E-04	175	SLC25A3	7.2E-04	275	SLC35C2	1.5E-03
76	TXNIP	2.1E-04	176	CABLES2	7.3E-04	276	VPS18	1.5E-03
77	KCTD17	2.2E-04	177	FOS	7.3E-04	277	SLC4A3	1.5E-03
78	TBC1D2B	2.2E-04	178	CBWD2	7.5E-04	278	SNAI2	1.5E-03
79	IFITM3	2.3E-04	179	NF1P8	7.5E-04	279	C6orf89	1.5E-03
80	EXOC3	2.4E-04	180	CHODL	7.5E-04	280	POLR2A	1.6E-03
81	CRTC1	2.4E-04	181	NME1	7.6E-04	281	CPS1	1.6E-03
82	SAMHD1	2.5E-04	182	GSTM2	7.6E-04	282	LIMK2	1.6E-03
83	3-Sep	2.5E-04	183	TRG-AS1	7.6E-04	283	PRR13	1.6E-03
84	CTNNB1	2.5E-04	184	ADGRA2	8.0E-04	284	TAGLN	1.6E-03
85	BCAS1	2.6E-04	185	CNBP	8.0E-04	285	TMEM170B	1.6E-03
86	DUSP7	2.6E-04	186	DNASE1	8.1E-04	286	ABHD13	1.6E-03
87	SMPD1	2.7E-04	187	AMFR	8.2E-04	287	RASA3	1.6E-03
88	PNP	2.7E-04	188	SLC38A4	8.3E-04	288	PDCD7	1.6E-03
89	FAM168A	2.8E-04	189	CRABP2	8.3E-04	289	ST3GAL3	1.6E-03
90	IL21R	2.8E-04	190	SLC6A10P	8.4E-04	290	ZFP64	1.7E-03
91	POMGNT2	2.8E-04	191	SLC25A4	8.4E-04	291	IDI1	1.7E-03
92	ARL8A	2.9E-04	192	FBLN1	8.4E-04	292	CDCA7	1.7E-03
93	IFITM2	3.0E-04	193	TPM4	8.5E-04	293	SSBP2	1.7E-03
94	MAT2B	3.0E-04	194	B4GALT3	8.5E-04	294	C11orf87	1.7E-03
95	PCDH9	3.1E-04	195	IFT57	8.6E-04	295	BBS12	1.7E-03
96	SGPP1	3.1E-04	196	VARS2	8.6E-04	296	TLE4	1.7E-03
97	SNCG	3.1E-04	197	CRABP1	8.6E-04	297	PCOLCE	1.7E-03
98	RRAGA	3.1E-04	198	DNMBP	8.8E-04	298	EIF2B2	1.7E-03
99	BZW2	3.2E-04	199	FENDRR	8.8E-04	299	STK24	1.7E-03
100	C2orf91	3.4E-04	200	CDH24	8.8E-04	300	CAPZB	1.7E-03
